# Extensive metagenomic analysis of the porcine gut resistome to identify indicators reflecting antimicrobial resistance

**DOI:** 10.1186/s40168-022-01241-y

**Published:** 2022-03-04

**Authors:** Yunyan Zhou, Hao Fu, Hui Yang, Jinyuan Wu, Zhe Chen, Hui Jiang, Min Liu, Qin Liu, Lusheng Huang, Jun Gao, Congying Chen

**Affiliations:** State Key Laboratory of Pig Genetic Improvement and Production Technology, Jiangxi Agricultural University, Nanchang, 330045 China

**Keywords:** Antimicrobial resistance gene, Rearing modes, Bacteria, Indicators, Pigs

## Abstract

**Background:**

Antimicrobial resistance (AMR) has been regarded as a major threat to global health. Pigs are considered an important source of antimicrobial resistance genes (ARGs). However, there is still a lack of large-scale quantitative data on the distribution of ARGs in the pig production industry. The bacterial species integrated ARGs in the gut microbiome have not been clarified.

**Results:**

In the present study, we used deep metagenomic sequencing data of 451 samples from 425 pigs including wild boars, Tibetan pigs, and commercial or cross-bred experimental pigs under different rearing modes, to comprehensively survey the diversity and distribution of ARGs and detect the bacteria integrated in these ARGs. We identified a total of 1295 open reading frames (ORFs) recognized as antimicrobial resistance protein-coding genes. The ORFs were clustered into 349 unique types of ARGs, and these could be further classified into 69 drug resistance classes. Tetracycline resistance was most enriched in pig feces. Pigs raised on commercial farms had a significantly higher AMR level than pigs under semi-free ranging conditions or wild boars. We tracked the changes in the composition of ARGs at different growth stages and gut locations. There were 30 drug resistance classes showing significantly different abundances in pigs between 25 and 240 days of age. The richness of ARGs and 41 drug resistance classes were significantly different between cecum lumen and feces in pigs from commercial farms, but not in wild boars. We identified 24 bacterial species that existed in almost all tested samples (core bacteria) and were integrated 128 ARGs in their genomes. However, only nine ARGs of these 128 ARGs were core ARGs, suggesting that most of the ARGs in these bacterial species might be acquired rather than constitutive. We selected three subsets of ARGs as indicators for evaluating the pollution level of ARGs in samples with high accuracy (*r* = 0.73~0.89).

**Conclusions:**

This study provides a primary overview of ARG profiles in various farms under different rearing modes, and the data serve as a reference for optimizing the use of antimicrobials and evaluating the risk of pollution by ARGs in pig farms.

Video abstract

**Supplementary Information:**

The online version contains supplementary material available at 10.1186/s40168-022-01241-y.

## Background

Antimicrobials are widely used in domestic animal husbandry for treating diseases, improving health, and promoting growth [1]. It is estimated that food-producing animals consume about 73% of the antimicrobials produced worldwide [2]. The extensive application of antimicrobials has facilitated the emergence of antimicrobial resistance (AMR) for microbes under selection pressure. Investigations have shown that long-term use of antimicrobials can result in the alteration of the composition and diversity of intestinal commensal microorganisms, thereby adversely affecting host health [3, 4]. The resistance genes can be transferred between humans and animals, and animal microbiota can thus serve as a reservoir of clinically important antimicrobial resistance genes (ARGs) [5]. Consequently, AMR is a potential risk to both the animals and humans. ARGs are now regarded as an emerging environmental pollutant, and they have attracted worldwide attention. The World Health Organization has recognized the antimicrobial resistance crisis as one of the most pressing threats to modern health care [6]. However, the origin, distribution, diversity, and transfer of AMR have not yet been clarified, and large-scale quantitative data concerning ARGs in the guts of animals are still lacking. The characterization of the resistome and quantifying the present level of ARGs are fundamental for evaluating the risk of AMR to public health. Moreover, different bacteria show distinct responses to antimicrobials, and different antimicrobials have different effects on microbial activity, composition, and metabolism [7]. Therefore, to avoid the utilization of broad-spectrum antimicrobials as far as possible, a comprehensive understanding of ARGs harbored in bacteria and the dynamics of gut microbiota caused by antimicrobials is needed to optimize the use of antimicrobials.

Some studies have already shown that the resistome is country-dependent due to country-specific farm systems and antibiotic supplementation [8, 9]. China is the largest producer of pigs with many different rearing modes and also the largest consumer of veterinary antimicrobials in the world and as such represents the largest hotspot of antimicrobial resistance. The contribution of China in the fight against global AMR is crucial [2, 10]. A recent survey reported that pigs accounted for 52.2% of the total antimicrobial usage in China [11]. ARGs that are transferred through ecological circulation have become an increasing threat to public health. Pork is one of the main consumer meat products, and the inappropriate use of antimicrobials in pig production is thought to contribute to AMR in humans [12]. However, systematic research on the resistome in pigs is lacking.

A previous study used quantitative PCR (qPCR) to analyze ARGs of 36 manure samples in three Chinese swine farms [13]. However, the throughput of qPCR is limited and specific primers from known sequences of ARGs are required. Metagenomic sequencing is a high-throughput method that can monitor the diversity and abundance of resistance genes quickly and extensively. Another study investigated ARGs in 287 pig fecal samples from France, Denmark, and China and compared the abundance of ARGs among countries [8]. A recent study analyzed the resistome of pig feces from four different industrialized feedlots in four distant provinces across China. The samples from each feedlot were pooled with feces sampled from three to five pigs [14]. However, all these surveys used a limited number of samples, and all pigs were raised in standard houses utilizing antimicrobials and having high stocking density. The distribution of the resistome in feces of pigs not under directly antibiotic selective pressure is completely unknown. All these studies were performed using fecal samples. It is not fully understood whether the fecal resistome can accurately reflect the characterization of the resistome in the intestinal lumen. However, a previous study reported different impacts of antimicrobials in the lumen microbial composition at various gut locations [15]. Previous studies in humans and cattle suggested that the composition of ARGs is altered at different ages [16, 17]. Thus, the age and gut locations need to be considered in investigating the composition of ARGs in humans and animals. Moreover, most of the previous studies only focused on the characterization of the resistome, including diversity, abundance, and drug resistance classes of ARGs, while the association between ARGs and gut microbiota, the main host microbes, and the risk assessment of resistance genes in pigs remain largely unexplored.

In this study, to characterize the composition and distribution of swine gut resistome in various farms under different rearing modes, we used feces samples of 425 adult pigs from three provinces of China. These experimental pigs were comprised of six wild boars captured from the wild, 21 Tibetan pigs raised in semi-free range conditions from three high-altitude farms, and 398 commercial or cross-bred experimental pigs housed in five standard farms with high stocking density (Additional file 2: Table S1). A comprehensive scan was performed to characterize swine gut resistome profiles using deep metagenomic sequencing. The bacterial species integrated ARGs were identified. We also compared the AMR between two growth stages and between cecum lumen and feces samples. Furthermore, to establish a simple and efficient method predicting the environmental risk of AMR and the pollution level of ARGs, we selected three subsets of resistance genes that can be used as indicators.

## Methods

### Animals and sample collection

All details about experimental pigs and sample collection were described in our previous study [18]. In this study, 12 samples from wild boars and 447 samples from domestic pigs were used. Briefly, 447 samples were harvested from 427 pigs raised in eight farms, including 427 fecal and 10 cecum lumen samples from adult pigs (142–951 days of age) and 10 fecal samples from piglets at 25 days of age (Additional file 2: Table S1). The eight farms were located in different counties of three provinces in China. Eight fecal samples from an F6 mosaic pig population were identified as outliers in the composition and abundance of ARGs and were excluded from further analysis. We also collected both fecal and cecum lumen samples from six wild boars to evaluate the level of ARGs in wild conditions (Additional file 2: Table S1). Based on the rearing modes including housing, feeds, and farm conditions, the experimental pigs were divided into three groups: free-range living, semi-free ranging, and standard farm housed. Wild boars (free-range living) were caught in the mountains near Nanchang, Jiangxi Province, China. Tibetan pigs (except NC-Tibetan pigs) were raised in three high-altitude farms (1400 m, 3480 m, and 3800 m) in Kangding, Sichuan Province, under semi-free ranging conditions with no commercial formula diets. The other experimental pigs were raised in five standard farm houses (NC-Tibetan, NC-F6, Dingnan, Jiangying, and Shahu) with commercial formula diets. This group included both commercial and cross-bred experimental pigs. No detailed information about the use of antimicrobials was provided for these experimental pigs. No wild boars received any antimicrobial treatments. Through consulting the farmers, we knew that Tibetan pigs raised in three farms of Kangding received less antimicrobial treatment than the pigs fed in standard farm houses. The latter that lived in pens at high density were treated with antimicrobials when they were sick. Antimicrobials were occasionally added to the diet. Six Tibetan pigs raised in the NC-Tibetan farm were bought from a farm in Gansu province, where they were fed under the same program as in the Kangding farm. These six Tibetan pigs were transported to a farm in Nanchang and raised with a commercial formula diet under the program for commercial pigs for 2 years before sampling. The 16 cecum lumen samples from wild boars (*n* = 6) and pigs in NC-F6 farms (*n* = 10) were harvested from the middle part of the cecum within 30 min after slaughter. To investigate the ARGs in piglets, we collected 10 fecal samples from the piglets at the NC-F6 farm. All samples were kept in liquid nitrogen during transportation and then stored at −80°C until use.

### DNA extraction, library construction, and metagenomic sequencing

Microbial DNA was extracted using a QIAamp Fast DNA Stool Mini Kit (Qiagen, Germany) according to the manufacturer’s instructions. DNA concentration and quality were monitored using a Qubit 2.0 Fluorometer (Life Technologies, USA) and 0.8% agarose gels electrophoresis. The libraries for metagenomic sequencing were constructed using NEBNext® Ultra™ DNA Library Prep Kit following the manufacturer’s instructions (NEB, USA). Briefly, qualified DNA samples were randomly fragmented to a size of about 350bp by sonication. The DNA fragments were end-polished, and an A-tail was added and linked to a sequencing adaptor. After cluster generation following the manufacturer’s protocol, all library preparations were sequenced on a Novaseq 6000 platform using a paired-end sequencing strategy (Illumina, USA).

### Bioinformatic analysis of metagenomic sequencing data

Metagenomic sequence reads were filtered to exclude adapter and low-quality sequences using fastp (v0.19.4) with options “--cut_by_quality3 -W 4 -M 20 -n 5 -c -l 50 -w 3” [19]. The reads that matched the host genomic DNA sequence (Sscrofa11.1) by BWA MEM (v0.7.17-r1188) [20] were removed. All clean sequence reads were assembled for each sample individually by MEGAHIT (v1.1.3) with kmer values from 27 to 87 [21]. The clean sequence reads were aligned to the assembled contigs using Bowtie 2 (v2.3.4.1) [22] to acquire unassembled reads of each sample. All unassembled reads of the experimental samples were clustered together and co-assembled using MEGAHIT (v1.1.3) with the same parameters. The contigs with lengths greater than 500 bp were used for gene (open reading frame (ORF)) prediction by MetaGeneMark (v3.38) [23]. Redundant genes were removed by CD-HIT (v4.7) at the 95% identity and 90% coverage of protein sequences [24]. The genes with length less than 100bp and the numbers of mapped reads < 2 in all samples were filtered out [25–27]. The resulted non-redundant genes in the catalogue were aligned to the NCBI NR database (version: 2019-04) using DIAMOND (v0.9.24) [28] at the *e*-value = 1e−5 that is the threshold commonly used in many other studies [29–31]. Taxonomic annotations of genes were determined based on the lowest common ancestor algorithms by BASTA (v1.3.2.3) at the thresholds of the matched sequence length > 25 bp, identity >50%, and the annotation shared by at least 60% of hits [32]. Those genes annotated to Eukaryota (except fungi) were excluded from further analysis. ARGs were identified by alignment of non-redundant genes against the Comprehensive Antibiotic Resistance Database (CARD) using RGI (v4.2.2) with the option “main -a diamond -t protein” [33].

### Estimation of the abundances of genes, bacterial taxa, and ARGs

Gene abundance was quantified by mapping clean reads to the gene catalogue using BWA MEM (v0.7.17-r1188) and computing the counts of mapped reads of each gene in each sample by featureCounts (v2.0.1) [20, 34]. The gene abundance was normalized to FPKM by the formula:

$$\mathrm{FPKM}=\frac{numFragments\ }{\frac{geneLength}{1000}\times \frac{totalNumReads}{\mathrm{1,000,000}}},$$

where *numFragments* represents the number of fragments mapped, *geneLength* is the length of the corresponding gene, and *totalNumReads* represents the total number of fragments per sample.

The abundances of microbial taxa and ARGs were calculated by summing the abundances of all members in each category (microbial taxa or ARGs). The sum of the abundances of all resistance genes in each sample was calculated to assess the total AMR level [9, 13]. The total number of all types of ARGs in each sample was defined as the richness of ARGs [35]. We also summed the abundance of ARGs in each AMR class (drug level) and antimicrobial resistance mechanism as the abundance of each of these items. The mean value of the abundances of each resistance gene, AMR class, resistance mechanism, and bacterial species in all pigs of each population was treated as the abundance of these items in that population.

### Identification of the bacterial species integrated ARGs

The procedures for identifying the carriers of ARGs are shown in Additional file 1: Fig. S14. In brief, the protein sequences in the non-redundant gene catalogue were aligned to CARD and NCBI NR databases using the methods described above. If a protein sequence was simultaneously annotated to an ARG in the CARD database and a microbial taxon in the NCBI NR database, we considered that this microbial taxon was the carrier of the corresponding ARG. Multiple protein sequences might be annotated to one type of resistance gene or a microbial taxon because the non-redundant gene catalogue was generated at 95% identity of the protein sequence. Distribution of ARGs in bacteria of different taxonomic levels was plotted as a Sankey diagram using the *networkD3* package (https://cran.r-project.org/web/packages/networkD3) [36] in R (v3.6.2). The distribution network between ARGs and their carriers was visualized in the Cytoscape platform (version: 3.5.1) [37]. The average abundance of a resistance gene in all samples represented the abundance of that resistance gene in a bacterial species (shown via the thickness of connecting lines in the network).

### PCoA and Procrustes analyses

The normalized abundance (FPKM) of ARGs and bacterial species were first Hellinger transformed and then used for principal coordinate (PCoA) analysis based on Bray-Curtis distance using the *vegan* package (https://cran.r-project.org/web/packages/vegan) [38] in R (v3.6.2). The “protest” function in the vegan package was used to analyze the Procrustes correlation between the bacteriome and the resistome.

### Selection of the indicators for evaluating the pollution level of ARGs

To accurately predict the total AMR level and richness of resistance genes using a subset of ARGs, we systematically tested different combinations of ARGs to identify a subset of resistance genes from all 349 ARGs that could be used to predict the ARG pollution level of environmental samples according to the method described by Bengtsson-Palme [35]. In brief, Spearman’s rank correlation between the abundance of each resistance gene and the total AMR abundance was first calculated for 425 fecal samples from adult pigs. The gene with the highest correlation coefficient was selected as the first candidate among the indicators. This first candidate was combined with each of the remaining ARGs, and the correlation between the sum abundance of each pair of genes and the total AMR abundance of the samples was calculated to find the combination of gene pairs with the highest correlation coefficient. At each iteration, a new gene was added to the subset to find the optimal combination. Selection of the indicators for predicting the richness of resistance genes was similar to that for abundance. For the indicators predicting both abundance and richness of ARGs, the combination of ARGs with the highest average value of correlation coefficient calculated for abundance and richness was selected at each iteration. A subset of ARGs was considered to be potential indicators when the *P* value was below 0.05. Cumulative prediction power (Spearman’s rank correlation between the abundance of ARGs in a subset and the total abundance or richness of ARGs in the tested sample following the increased number of indicators) was visualized using the *ggplot2* package (https://cran.r-project.org/web/packages/ggplot2/) [39] in R (v3.6.2). To test the prediction power of the selected indicators for the abundance and richness of resistance genes in fecal samples of pigs, we downloaded the abundance matrix of resistance genes from a study of European pig feces [9]. We summed the abundance of ARGs in each sample at the level of the ARG category and then calculated the cumulative prediction power for total AMR levels and the richness of ARGs in the downloaded dataset. The indicators that were included in the selected subset but not detected in the downloaded dataset were excluded from the prediction analysis.

### Other statistical analyses

The effect of gender on the abundance and richness of ARGs in the guts of pigs was analyzed for each of the seven farms. Wild boars were excluded from this analysis because their genders were unknown. The effect of host genetics on the richness and abundance of ARGs was tested in two pig breeds from Dingnan farm, Licha and a hybrid line of Berkshire × Licha. The comparison of richness and abundance of ARGs in pigs between 25 and 240 days of age was performed in ten pigs from a mosaic population raised in an NC-F6 farm. The comparison of the ARGs between cecum lumen and fecal samples was performed in six wild boars and ten pigs from the NC-F6 farm at the age of 240 days. All comparisons described above were performed using the Wilcoxon test in the *ggpubr* package [40] in R. Multiple tests were corrected and false discovery rate (FDR) < 0.05 was considered as the significance level. Core resistome was defined as those ARGs present in at least 95% of individuals [9]. The *vegan* package in R (v3.6.2) was used to compute the α-diversity of gut microbiota measured via the number of observed species, Simpson’s index (1-D), and Shannon’s index. All heatmaps were plotted using the *pheatmap* package in R (v3.6.2).

## Results

### The diversity, abundance, and drug resistance classes of fecal resistome in tested samples

Deep metagenomic sequencing of 433 fecal samples from adult pigs identified a total of 1295 ORFs recognized as antimicrobial resistance protein-coding genes by aligning against the CARD database. The ORFs were clustered into 349 unique types of ARGs (Additional file 3: Table S2) and could be further classified into 69 drug resistance classes based on the antimicrobials to which they conferred resistance (Additional file 4: Table S3). In order to exclude the effect of outliers on the statistical analysis, we filtered eight samples from an NC-F6 farm in which the total AMR levels exceeded the mean ±3 × SD (standard deviations) (Additional file 1: Fig. S1a), and the richness of gut microbiota (number of species) was significantly lower than that in other samples from the same population (Additional file 1: Fig. S1b). Furthermore, ARGs belonging to the drug resistance classes of aminoglycoside, M-L-S (macrolide-lincosamide-streptogramin), lincosamide, and phenicol were particularly enriched in these eight samples (Additional file 1: Fig. S1c). We suspected that the pigs that these samples were harvested from might have been given antimicrobials. Finally, 425 fecal samples from adult pigs were included in the following analyses.

**Fig. 1:**
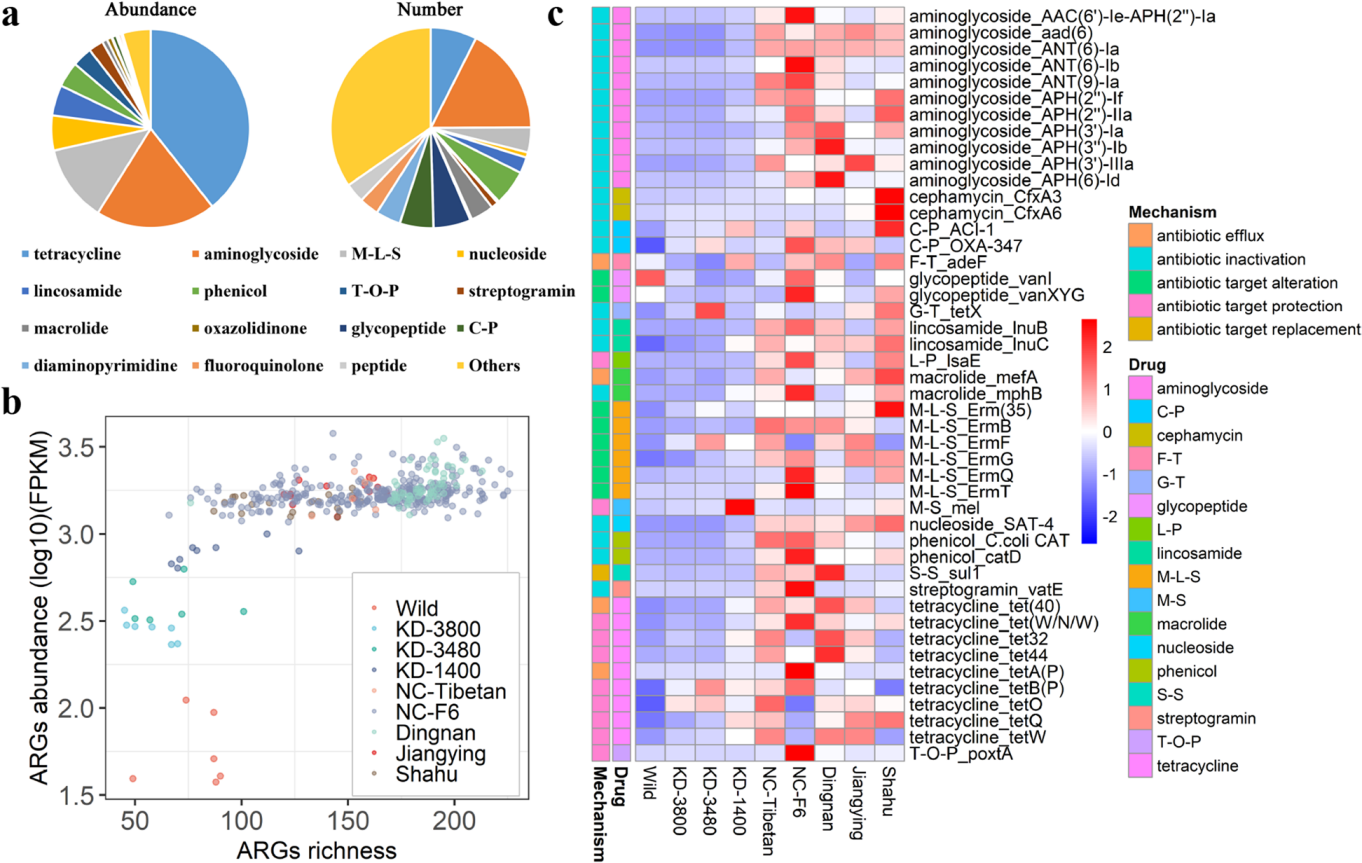
The composition, abundance, and richness of fecal resistome in pigs under different rearing modes. **a** The abundances and the numbers of antimicrobial resistance genes (ARGs) for each antimicrobial. All ARGs were classified according to the antimicrobial for which they showed resistance. The abbreviated names of antimicrobial classes were used in all figures, and the corresponding full names were presented in Additional file 4: Table S3. **b** The abundances and the numbers of ARGs in all samples (*n* = 425). All samples were divided into nine groups according to the farms that the samples were from. All wild boars were considered as one group. Wild represents wild boars; KD-3800, KD-3400, and KD-1400 represent Tibetan pigs raised in three high-altitude farms (3800m, 3400m, and 1400m) from Kangding; NC-Tibetan represents Tibetan pigs raised in Nanchang; NC-F6 represents pigs from a F6 mosaic population raised in Nanchang; Dingnan represents pigs raised in Dingnan; Jiangyin and Shahu represent Duroc pigs raised in Jiangyin and Shahu farms. More details of nine pig populations are shown in Additional file 2: Table S1. **c** The abundance of 46 core ARGs in each of nine pig populations, and their corresponding antimicrobial classes and resistance mechanisms. The ARGs existed in at least 95% samples (*n* ≥ 404) were defined as core ARGs in pig feces. Two color bars on the left of the heatmap represent the resistance mechanism and corresponding antimicrobial class of each resistance gene, respectively. The abundance of 46 core ARGs varied greatly in different pig groups

Tetracycline resistance was most enriched in pig feces, followed by aminoglycoside and M-L-S resistance classes (Fig. 1a). The abundance of ARGs in these three resistance classes accounted for 71.4% of the total abundance of resistance genes. The high abundance of these three resistance classes was observed in pigs raised in standard farms (NC-Tibetan, NC-F6, Dingnan, Jiangying, and Shahu farms). In addition, lincosamide and nucleoside resistance classes also had a higher abundance in the pigs raised in standard farms than in Wild boars or Tibetan pigs (in Kangding farms) (Additional file 1: Fig. S2). Among 69 resistance classes, aminoglycoside resistance contained the largest number of unique ARGs (61 ARGs) including *AAC*, *ANT*, and *APH* gene families, followed by the resistance classes of tetracycline (*tet*, 26 ARGs), glycopeptide (*van*), and phenicol (including chloramphenicol acetyltransferase and major facilitator superfamily antibiotic efflux pump gene families) and the resistance to both cephalosporin and penam (this class was abbreviated as C-P; most of the genes in this class belonged to the OXA beta-lactamase family) (Fig. 1a and Additional file 3: Table S2). Thirty drug resistance classes had a high abundance of ARGs, but there were few varieties of ARGs (Additional file 1: Fig. S3). Accordingly, some samples with high abundance of resistance genes might have low richness of resistance genes, whereas the samples with high richness of resistance genes always had a high abundance of resistance genes (Fig. 1b). The major resistance mechanisms of ARGs identified in this study were antibiotic inactivation, antibiotic target alteration, antibiotic target protection, and antibiotic efflux (Additional file 1: Fig. S4).

The prevalence and the average abundance of all ARGs in the tested samples are presented in Additional file 1: Fig. S5. The ARGs present in at least 95% of the tested samples were defined as core ARGs. A total of 46 core ARGs were identified in the fecal samples. The ARGs whose abundance was listed in the top five were *tet(W/N/W)*, *APH(3')-IIIa*, *tet(40)*, *ErmB*, and *tetQ*. Twelve ARGs showed low prevalence (< 30%) but had a high abundance in the detected samples (Additional file 1: Fig. S5). The 46 core ARGs were mainly related to the resistance of aminoglycoside, tetracycline, and M-L-S, and they varied in abundance in different pig farms (Fig. 1c). Aminoglycoside resistance genes belonged to *ANT(6)*, *APH(2”)*, and *APH(3')* families. These ARGs confer resistance to aminoglycoside antimicrobials by the mechanism of antibiotic inactivation. Most tetracycline resistance-associated ARGs belong to the gene family of tetracycline-resistant ribosomal protection protein and exert the resistance effect through the mechanism of antibiotic target protection. The genes in the Erm 23S ribosomal RNA methyltransferase family confer resistance to M-L-S antimicrobials by the mechanism of antibiotic target alteration (Additional file 3: Table S2). The 46 core ARGs occupied 90% (on average) of the total abundance of all ARGs in 425 fecal samples from adult pigs (Additional file 1: Fig. S6).

### Rearing modes significantly affected the profiles of ARGs in pig feces

We first investigated the factors influencing the richness and abundance of ARGs. Gender had a limited effect on the abundance of fecal ARGs, although the difference was significant before being corrected for the FDR in Tibetan pigs from the KD-1400 farm (*P* = 0.030, FDR = 0.132) and Duroc pigs from the Shahu farm (*P* = 0.038, FDR = 0.132). Gender also showed no effect on the richness of fecal ARGs (Additional file 1: Fig. S7). As for the effect of host genetics, two pig breeds were raised in the Dingnan pig farm, Licha and a hybrid line of Berkshire × Licha. However, there were no significant differences in the abundance or richness between the two breeds (*P* > 0.05, Additional file 1: Fig. S8).

**Fig. 2:**
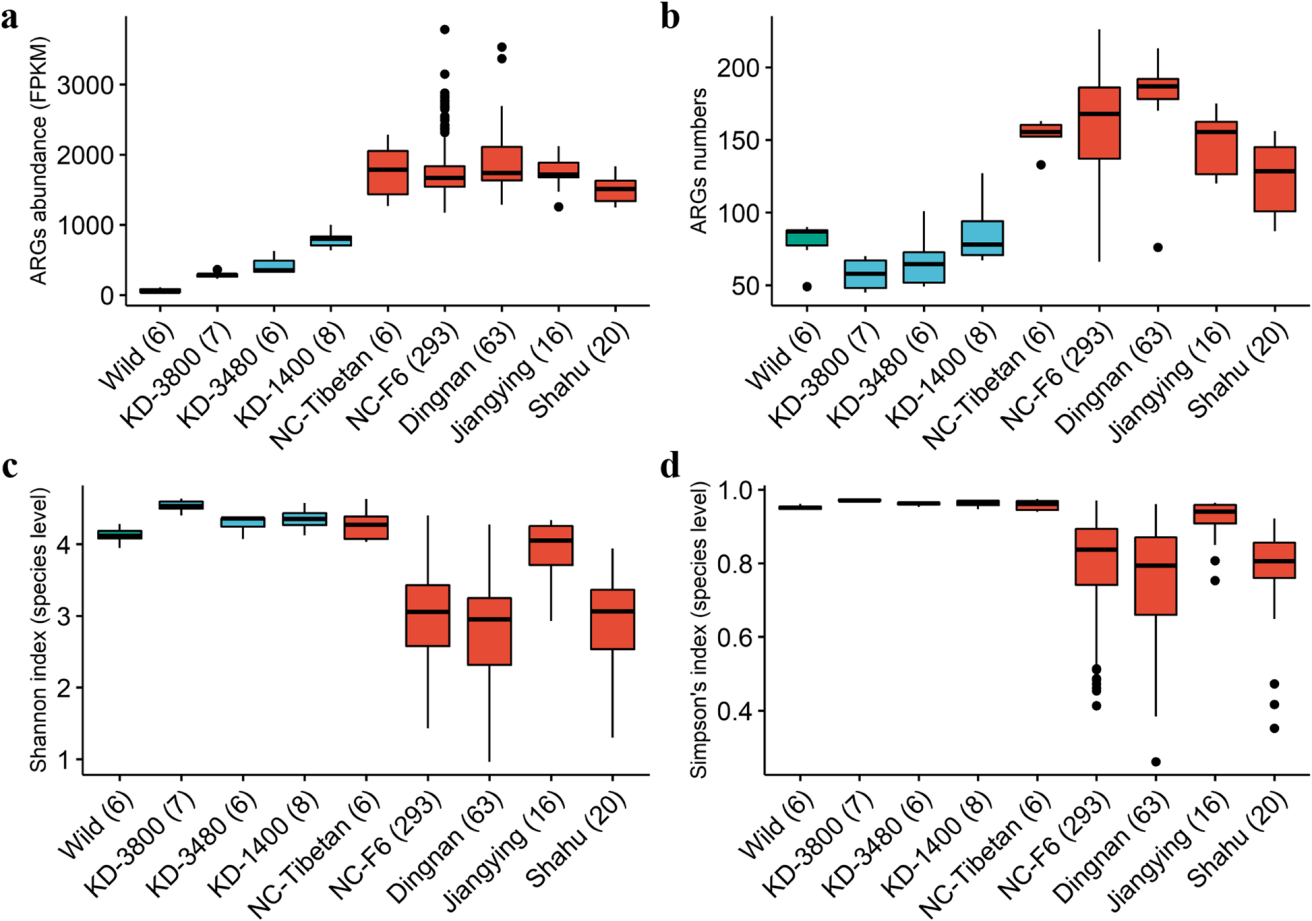
Comparisons of the abundances and the gene numbers of antimicrobial resistance genes (ARGs), and the α-diversity of bacterial composition among experimental pigs from nine populations. **a** Comparisons of the abundances of ARGs among nine populations. **b** Comparisons of the richness of ARGs among nine populations. **c** Comparisons of Shannon index of gut microbiota at the species level among nine populations. **d** Comparisons of Simpson’s index of gut microbiota among nine populations. The pigs showing the lower diversity (Shannon index and Simpson’s index) of gut microbiota tend to have higher ARG abundance and richness. The boxplots are colored according to the rearing modes (green: free-range living, blue: semi-free ranging, red: standard farm housing). Numbers in brackets of *x*-axes represent the sample number of each group

PCoA showed that the abundance and composition of ARGs in the fecal resistome varied in pigs under different rearing modes. Tibetan pigs raised in three farms with similar rearing modes in Kangding had the higher similarity of resistome composition. The composition of resistome varied widely among Wild boars, which may be explained by different living environments for these wild boars (Additional file 1: Fig. S9). And then, the pair-wise comparisons were performed among nine populations. Although the NC-F6 population had the largest sample size, pigs from the Dingnan farm had the highest abundance of ARGs, which was more than 31 times than that of wild boars (the average abundance: 1916 FPKM in pigs from the Dingnan farm vs. 62 FPKM in wild boars) (Additional file 5: Table S4). Tibetan pigs from each of three Kangding farms (KD-3800, KD-3480, and KD-1400) raised under semi-free ranging conditions with no commercial formula feed had a significantly lower abundance of ARGs than those pigs raised in standard farms (NC-Tibetan, NC-F6, Dingnan, Jiangying, and Shahu farms) (Fig. 2a, Additional file 5: Table S4 and Additional file 6: Table S5) (FDR < 0.05). The lowest abundance of ARGs was found in wild boars, animals that are rarely exposed to antimicrobials. However, wild boars had a higher richness of AMR genes than Tibetan pigs raised in semi-free ranging conditions (FDR < 0.05) (Fig. 2a, b). More importantly, significant differences in both abundance and richness of ARGs were observed among pigs from the same breed but from different farms, such as Duroc pigs from Jiangying and Shahu farms and Tibetan pigs from three high-altitude farms (1400 m, 3480 m, and 3800 m) and the Nanchang farm (NC-Tibetan) (FDR < 0.05). The composition and abundance of fecal resistance in Tibetan pigs from the Nanchang farm were similar to those in pigs from commercial farms, but significantly different from those of Tibetan pigs from three farms in Kangding (FDR < 0.05). This was mainly due to the different husbandry factors among pig farms, including feeds, the farm environment, and the use of antimicrobials. Thus, the farms with similar rearing modes tended to have similar levels of AMR. Interestingly, lower diversity (measured by Shannon’s index and Simpson’s index) of the gut microbiota was observed in those pigs having higher abundance and richness of ARGs (FDR < 0.05) (Fig. 2, Additional file 5: Table S4 and Additional file 6: Table S5).

### Comparison of resistome in fecal samples between two growth stages and between cecum lumen and feces

**Fig. 3:**
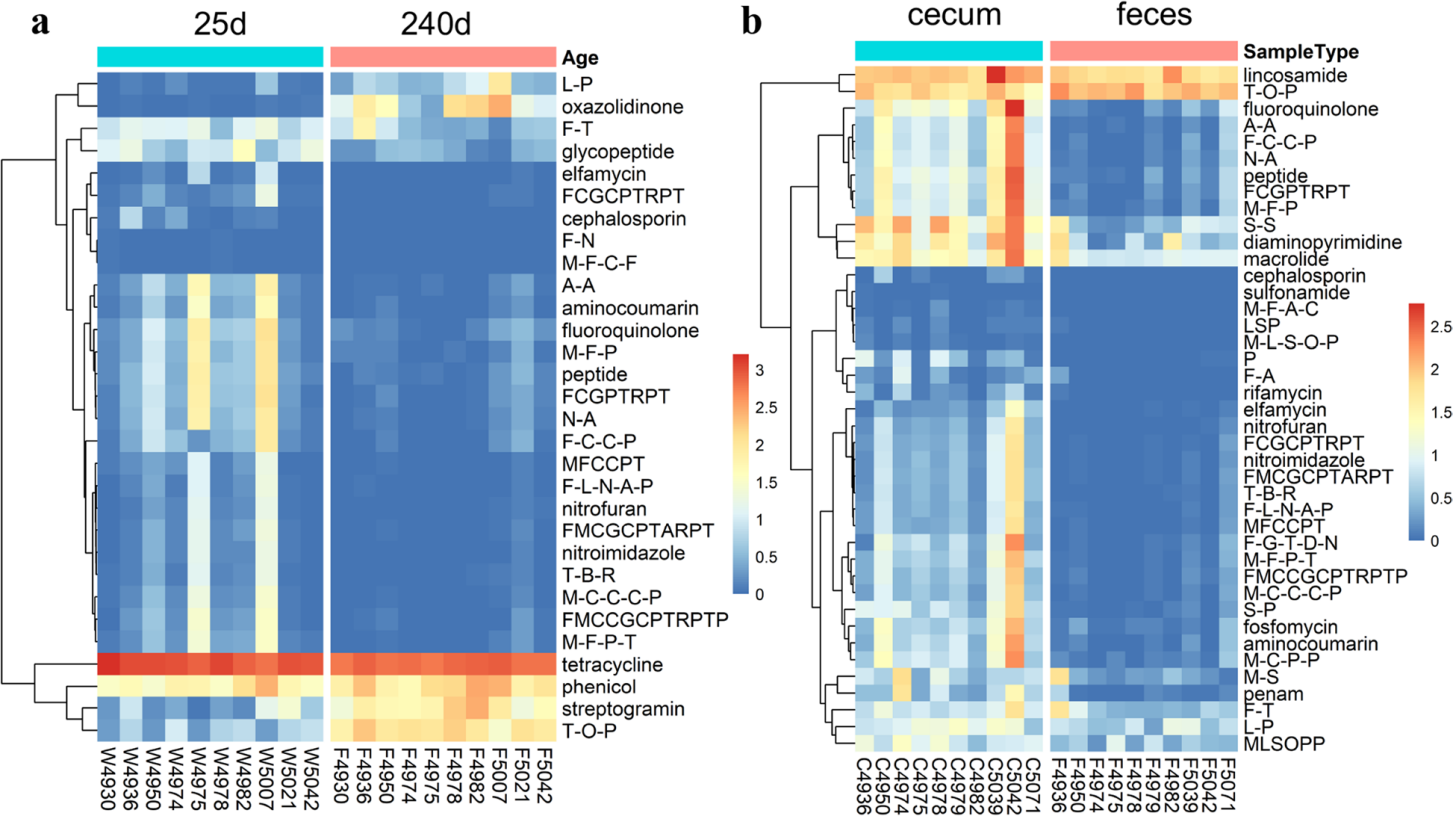
Comparison of antimicrobial resistance classes between 25 and 240 days of age and between cecum lumen and feces samples. **a** Comparison of antimicrobial resistance classes between 25 and 240 days of age in pigs from the NC-F6 population. **b** Comparison of antimicrobial resistance classes between cecum lumen and feces samples in pigs from the NC-F6 population. The *x*-axis shows the sample IDs. W + numbers: feces samples at the age of 25 days, F + numbers: feces samples at the age of 240 days, and C + numbers: cecum samples at the age of 240 days. The same number represents the same pig. The antimicrobial resistance genes (ARGs) were classified into antimicrobial resistance classes according to antimicrobials that they show resistance for (the *y*-axis). The abundance of all ARGs belonging to a antimicrobial resistance class in a sample was summed together as the abundance of AMR class in that sample and was used for further comparisons. The full names of antimicrobials are presented in Additional file 4: Table S3. The comparisons were performed using the Wilcoxon test in the ggpubr package in R, and the *P* value corrected for multiple tests (FDR < 0.05) was treated as the significance threshold

To compare the resistome profiles between different age groups, we used metagenomic sequencing data from ten pigs (NC-F6 population) of which fecal samples were collected at 25 and 240 days of age. The 46 core ARGs showed a high prevalence in fecal samples of both piglets and adult pigs (Additional file 1: Fig. S10). We then compared the fecal resistome profiles between 25 and 240 days of age in these ten pigs. Although there were no significant differences in either total abundance or the number of resistance genes between 25 and 240 days of age (Additional file 1: Fig. S11a, b), at the level of drug resistance classes, we identified 30 classes showing significantly different abundances (Additional file 7: Table S6; Wilcoxon test, FDR < 0.05). For examples, Oxazolidinone, T-O-P (tetracycline antibiotic, oxazolidinone antibiotic, and phenicol antibiotic), and streptogramin resistance classes were significantly enriched at the age of 240 days (FDR < 0.05), while tetracycline resistance was more abundant at the age of 25 days (FDR < 0.05) (Fig. 3a).

We next compared the resistome profiles between cecum lumen and fecal samples from the same individual in six wild boars and ten pigs from the NC-F6 farm. Similarly, the 46 core ARGs also had a high prevalence in cecum lumen samples (Additional file 1: Fig. S10). We did not observe the significant differences in either abundance or richness of resistance genes between cecum lumen and feces in wild boars that had not received any treatment with antimicrobials (Additional file 1: Fig. S11c). The same result was found at the level of drug resistance class. However, in pigs from the NC-F6 farm, the richness of ARGs in the cecum lumen was significantly higher than that in feces (FDR = 3.61 × 10^−4^, Additional file 1: Fig. S11d). We identified seven ARGs, *dfrG*, *RbpA*, *APH(3')-IIa*, *dfrA14*, *CTX-M-27*, *lsaB*, and PC1 beta-lactamase (*blaZ*), as being prevalent in cecal samples. However, these seven ARGs were not detected in fecal samples from the same individuals. These genes were also absent in wild boars (Additional file 1: Fig. S12). At the level of drug resistance classes, 41 resistance classes showed differential abundance between cecum lumen and fecal samples in pigs from the NC-F6 farm (Wilcoxon test, FDR < 0.05) (Fig. 3b and Additional file 7: Table S6), although there was no significant difference in total abundance of AMRs (Additional file 1: Fig. S11d). We further confirmed that this inconsistent result between wild boars and the pigs from the NC-F6 farm was reliable and not caused by different sample sizes (6 vs. 10 pigs) by randomly selecting six out of ten pigs from the NC-F6 farm for each of 1000 times of comparison analyses for the 41 resistance classes identified above (Additional file 1: Fig. S13).

### Bacterial species carrying ARGs

**Fig. 4:**
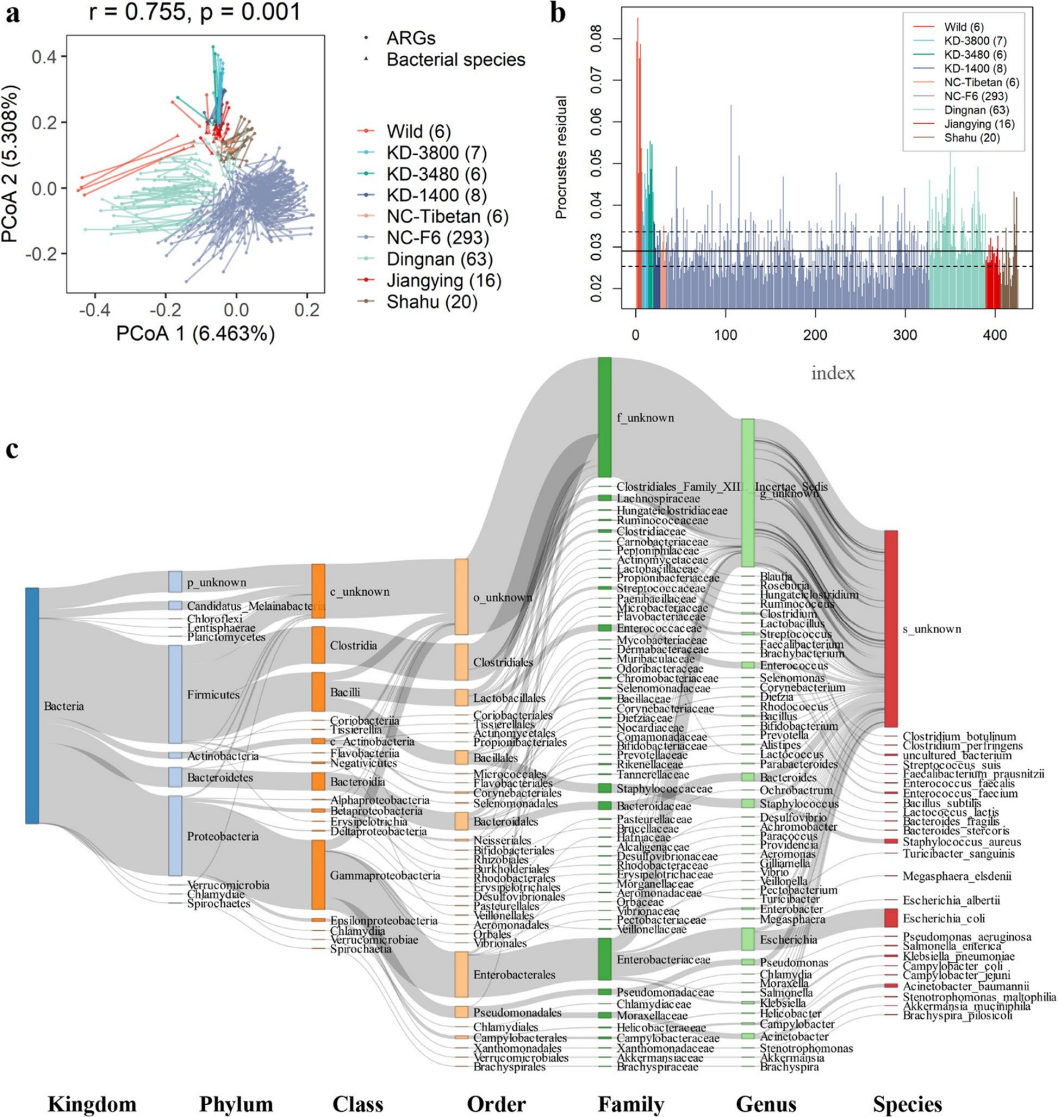
Correlation of antimicrobial resistance genes (ARGs) with gut bacteria. **a** The association in the abundances between gut bacterial species and ARGs by Procrustes analyses. The dots show the ordination positions of the abundances of bacterial species in each sample, and the triangulars indicate the ordination positions of the abundances of ARGs. The length of lines between dot and triangular shows the Procrustes residuals. **b** Significantly different degrees of the correlationships in abundances between bacterial species and ARGs among nine pig populations. Horizontal lines indicate the median (solid), 25% and 75% quantiles (dashed) of Procrustes residuals. The *y*-axis shows Procrustes residuals and the *x*-axis indicates the number of samples. For **a** and **b**, the numbers in brackets of the graphic symbol represent the sample number of each population. **c** The distribution of 1295 antimicrobial resistance protein-coding genes on bacteria at different taxonomy levels. The colors of the rectangles represent different taxonomy levels. The length of the rectangles indicates the number of ARGs

We first tested the correlation between ARGs and bacterial taxa using Procrustes analysis. Overall, the composition of the resistome was significantly correlated with the bacterial composition (*r* = 0.755, *p* = 0.001) (Fig. 4a). Most of the variation in fecal resistome between farms seemed to be explained by the variation in the bacteriome (Fig. 4a). However, the strength of the correlation between the bacteriome and the resistome depended on the farm. For example, in Tibetan pigs from the KD-3800 and KD-3480 farms and especially in wild boars, the ordinations of resistome and bacteriome were very dissimilar, suggesting that the resistomes in these pigs were not strongly associated with the bacteriome. However, the ordinations of bacteriome and resistome were more similar in pigs from standard commercial farms (Fig. 4b). This was probably due to the reasons of lesser utilization of antimicrobials and lower abundance and richness of ARGs that might be from environments (FDR < 0.05, Fig. 2) in wild boars and in pigs from Kangding farms.

**Fig. 5:**
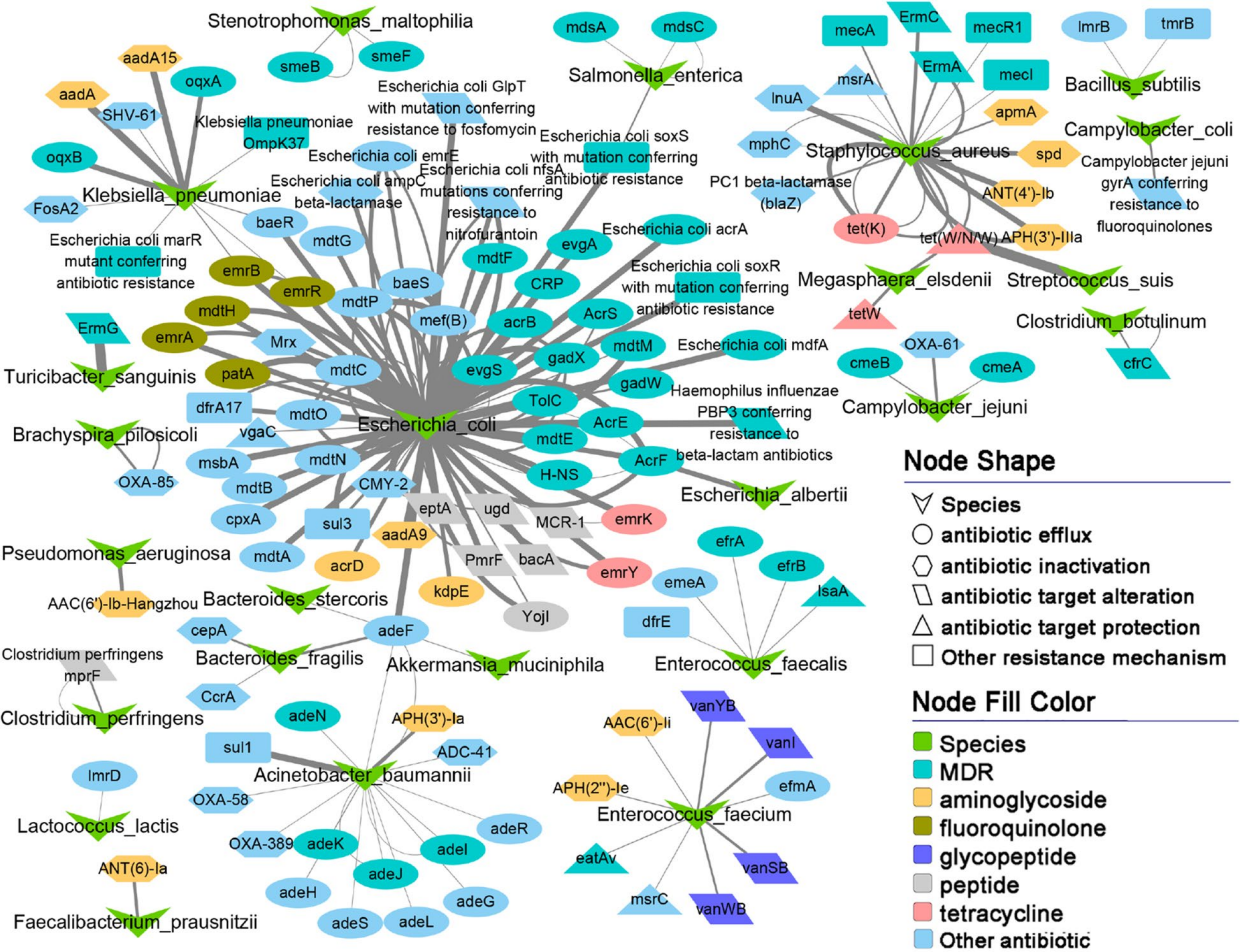
Carriers of antimicrobial resistance genes (ARGs) at the species level. Twenty-four bacterial species carrying 128 types of ARGs and the relationship between carriers and ARGs. The thickness of each connection line (edge) between two nodes represents the abundance of a resistance gene (ORF) on the bacterial species carrying it. If a resistance gene has the nature resistance to three or more antimicrobial classes, it was considered as a multidrug resistance gene (MDR, nodes with light green in network). Different colors and shapes of nodes indicate the antimicrobials that ARGs show resistance to, and resistance mechanisms

The 1295 ORFs annotated as ARGs were aligned to the NCBI NR database (version: 2019-04) to trace the bacteria possibly integrated ARGs (Additional file 1: Fig. S14). ARGs were widely distributed among various bacteria, particularly bacteria from Firmicutes (mainly from Clostridia and Bacilli) and Proteobacteria (mainly from Gammaproteobacteria). We also found that several bacteria from Bacteroidetes were also integrated ARGs. Enterobacterales, Clostridiales, Lactobacillales, Bacillales, Bacteroidales, and Pseudomonadales were the dominant orders harboring ARGs, and *Escherichia* was the bacteria integrated the largest number of resistance genes (Fig. 4c). Only 194 out of these 1295 ORFs could be linked to bacterial species. These 194 ORFs were assigned to 128 ARGs and were integrated in 24 bacterial species. *Escherichia coli* harbored the largest number of ARGs (96 out of 194 ORFs), of which 55 resistance genes conferred resistance to at least one of fluoroquinolone, peptide, and macrolide antibiotics (Additional file 8: Table S7). Furthermore, most of these 96 ORFs showed high abundance (Fig. 5). The 24 bacterial species had a high prevalence in the experimental samples (core bacterial species) (Additional file 9: Table S8). If 128 ARGs were the constitutive components of these 24 bacteria, they should also show high prevalence in the tested samples. However, only nine ARGs were observed in the list of core ARGs. This result suggested that most of the ARGs might be acquired rather than constitutive. Some pathogens were the major bacterial species integrated ARGs, including *Escherichia coli*, *Staphylococcus aureus*, *Enterococcus fecalis*, *Enterococcus faecium*, *Acinetobacter baumannii*, and *Klebsiella pneumoniae*. These bacteria are also known as multidrug-resistant bacteria. *Escherichia coli* especially shows resistance to nearly all antibiotics (Fig. 5). Some ARGs in *Escherichia coli*, such as *TolC*, *AcrS*, *soxR*, *acrA*, *soxS*, and *acrB*, have been reported (CARD database) [41] to be resistant to more than eight antibiotics (Fig. 5 and Additional file 3: Table S2). Notably, several bacteria such as *Akkermansia muciniphila* [42, 43], *Fecalibacterium prausnitzii* [44, 45], *Lactococcus lactis* [46], *Bacteroides fragilis* [47], and *Bacillus subtilis* [48, 49] that have been considered as probiotics or next-generation probiotics were also identified as carrying ARGs in their genomes. However, the number of ARGs integrated in these bacteria was significantly lower than that in other bacteria (Fig. 5). Some ARGs were discovered in multiple bacterial species. For example, *adeF* that was identified as a core ARG conferring multidrug resistance in *Acinetobacter baumannii* [50] was also identified in *Escherichia coli*, *Bacteroides stercoris*, *Akkermansia muciniphila*, and *Bacteroides fragilis*. The *tet(W/N/W)*, another core gene and a new tetracycline resistance gene (TRG) that was identified in a Chinese pig manure sample [51], was also detected in the genomes of *Staphylococcus aureus*, *Streptococcus suis*, and *Megasphaera elsdenii* (Fig. 5).

### Selection of indicators for evaluating the pollution level of ARGs in pigs

**Fig. 6:**
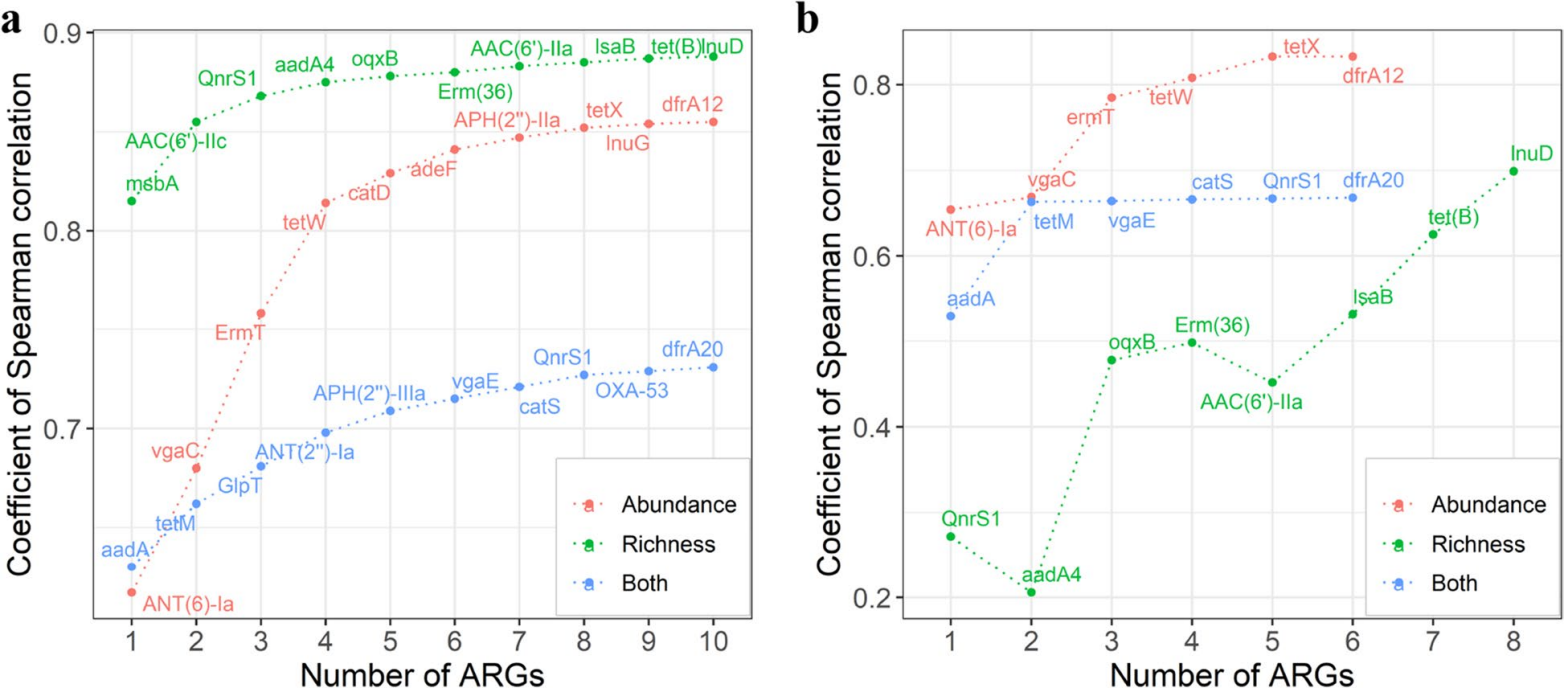
Indicators for evaluating the pollution level (abundance, richness, or both) of antimicrobial resistance genes (ARGs). **a** The distribution curves of Spearman’s rank correlation coefficients calculated from the correlation analysis between the abundances of selected indicators and all ARGs in the tested samples (red curve), between the abundances of selected indicators and richness of ARGs (number of ARGs) (green curve), and between the abundances of selected indicators, and both richness and abundance of all ARGs in the tested samples (blue curve) following the numbers of ARGs selected. **b** Validation of the prediction accuracy of AMR pollution using the selected ARG subsets using the dataset of 185 fecal samples downloaded from the public database (Munk et al. [9]). The red, green, and blue curves represent the distribution of correlation coefficients for abundance, richness, and both abundance and richness, respectively, as described above

To accurately predict the pollution level of resistance genes in pigs through a small subset of resistance genes, we optimized a subset of ARGs (see the “Methods” section) as the indicators for predicting the abundance and richness of ARGs in samples. First, we selected the indicators predicting the total AMR level that should be primarily related to the abundance of ARGs but not to diversity in the environment [52]. When *ANT(6)-Ia*, *vgaC*, *ErmT*, *tetW*, *catD*, *adeF*, *APH(2”)-IIa*, *tetX*, *lnuG*, and *dfrA12* were chosen, the correlation coefficient between the abundance of all ten selected ARGs and the total abundance of ARGs reached 0.85. Using only *ANT(6)-Ia*, *vgaC*, *ErmT*, and *tetW*, the correlation coefficient between the sum abundance of these four genes and the total abundance of all ARGs reached 0.81, suggesting the high accuracy of prediction using these genes (Fig. 6a). Among the selected genes, three genes (*tetW*, *adeF*, and *tetX*) are resistance genes for tetracycline; two genes (*ANT(6)-Ia* and *APH(2”)-IIa*) are aminoglycoside resistance genes, and *ErmT* is an M-L-S resistance gene (Additional file 10: Table S9). The *adeF* had the largest number (*n* = 173) of homologous proteins (ORFs) in the gene catalogue, and these were widely distributed in different bacteria (Additional file 11: Table S10). It was noted that the selected indicators had a high prevalence in the tested samples, ranging from 55.5 to 100%. Their abundances varied greatly in the samples (average abundance from 1.146 to 21.953 FPKM) (Additional file 10: Table S9 and Additional file 1: Fig. S15). This would explain the high prediction accuracy for the total abundance of ARGs in samples.

We then chose several ARGs as indicators for evaluating the richness of resistance genes in the tested samples. We selected ten ARGs related to aminoglycoside resistance (*AAC(6')-IIc*, *aadA4* and *AAC(6')-IIa*), M-L-S resistance (*Erm(36)*), tetracycline resistance (*oqxB* and *tet(B)*), and four other resistance classes (*msbA*, *QnrS1*, *lsaB*, and *lnuD*) (Additional file 10: Table S9). These indicators varied greatly in prevalence, ranging from 4.9 to 83.1%, but showed a low variation in abundance (average abundance from 0.005 to 0.735 FPKM) (Additional file 10: Table S9). Of note, seven out of these ten ARGs were present in Gammaproteobacteria (Additional file 11: Table S10), a bacterial class that harbored the most ARGs (Fig. 4c). We found that the correlation coefficient between the abundance of all ten selected ARGs and the richness of ARGs in the tested samples reached 0.89. Interestingly, when only *msbA* was used, the abundance of this gene was significantly correlated with the richness of ARGs in the tested samples, with a correlation coefficient of 0.82, suggesting that the richness of ARGs in a sample could be predicted through measuring only the abundance of *msbA*. We further selected a subset of ten ARGs that could be used to predict both abundance and richness of ARGs in the tested samples. These were *aadA*, *tetM*, *GlpT*, *ANT(2”)-Ia*, *APH(2”)-IIIa*, *vgaE*, *catS*, *QnrS1*, *OXA-53*, and *dfrA20* (Fig. 6a). These 10 ARGs were related to the resistance of aminoglycoside (*aadA*, *ANT(2”)-Ia* and *APH(2”)-IIIa*), tetracycline (*tetM*), and six other resistance classes (Additional file 10: Table S9). The average correlation coefficient between the total abundance of these selected indicators and the abundance and richness of ARGs in the tested samples was 0.73, implying that these ARGs could be used to predict both the abundance and richness of AMR genes in samples (Fig. 6a).

To further verify the performance of these selected ARGs as indicators for predicting total abundance and/or richness of ARGs, we used an ARG dataset from 185 samples across 181 pig herds from nine European countries [9] as a validation dataset. For the indicators used to predict the total abundance of ARGs, only six (*ANT(6)-Ia*, *vgaC*, *ErmT*, *tetW*, *tetX*, *dfrA12*) out of these ten indicators were identified in this validation dataset. Interestingly, a high correlation coefficient (*r* = 0.83) was obtained between the total abundance of six indicators and the abundance of all ARGs in the validation dataset (Fig. 6b). Similar results were observed for the indicators used to predict richness (*r* = 0.71, *p* = 1.52E−29) and to predict both abundance and richness (average *r* = 0.67, average *p* = 1.35E−16) of ARGs in the validation dataset (Fig. 6b).

## Discussion

The level of antimicrobial resistance varies greatly between countries, regions, and even individual herds [8–10, 14, 16, 53]. In this study, we used 425 fecal samples of adult pigs from wild boars and from pigs in eight farms in different regions of China to characterize the composition, prevalence, and abundance of fecal resistome in pigs under different rearing modes. To our knowledge, this is the first comparative study of ARGs between cecum lumen and feces and between two age groups.

Fecal resistome varied among farms. This may be driven by the different management factors including farm location, the use of antimicrobials, housing conditions, and feeding. The pigs raised under similar rearing modes showed similar abundance and richness of fecal ARGs (Fig. 2). In general, the majority of ARG classes in pig feces were those conferring resistance to tetracycline, aminoglycoside, and M-L-S. Tetracycline resistance is most common in pig farms worldwide, including farms in China, the USA, and Europe [8–10, 54]. This could be partially explained by the long-term use of tetracycline in pig production [55, 56].

We found that the drug resistance classes differed between two age groups. This might have been caused by factors such as diet, environment, and antimicrobial use. Research on the fecal resistome of dairy cattle has also indicated that the abundance of ARGs dynamically changed during nursing and was associated with diet [17]. A study of fecal antimicrobials in humans identified age-specific classes of antimicrobials among children, adults, and elderly people [16], suggesting that distinct ARG profiles at different ages may be associated with exposure to different antimicrobials at each age. From the comparison of resistome profiles between two gut locations in two farms under different rearing modes, we found that the abundance and diversity of resistance genes in the cecum lumen tended to be higher than that in feces, especially in pigs under antimicrobial selection pressure (NC-F6). This meant that it was not enough to evaluate the degree of AMR pollution only using fecal and environmental samples, and the problem of AMR pollution may be more serious than previously perceived.

Selective pressure of antimicrobials reduces the diversity of the microbiota, promotes the enrichment of drug-resistant bacteria, and further accelerates the rise of AMR levels [57–59]. However, we should note that higher diversity of the gut microbiota means a multitude of mechanisms for genetic mobility and more types of ARGs that are the sources of large numbers of unknown resistance genes having the potential for horizontal transfer [60]. This may explain why Tibetan pigs from the NC-Tibetan farm had a high abundance and diversity of ARGs (Fig. 2a, c). Tibetan pigs from the NC-Tibetan farm had been raised in Nanchang for 2 years. These pigs also had the highest M-L-S resistance level (Additional file 1: Fig. S2). This might be partially explained by horizontal gene transfer through mobile genetic elements among the strains of the same bacterial species or even among different species to adapt to the changes in the farm environments and diets as well as the selection pressure of antimicrobials [61]. Some ARGs were present in only a small number of samples but had high abundance (Additional file 1: Fig. S5), indicating that intra-individual transfer of resistance among microbiota occurred under specific selection pressure of antimicrobials [52]. We did not find a significant relationship between the richness and the abundance of ARGs (Fig. 1a, b). As demonstrated in a previous study, if mobile resistance genes had been transferred between bacteria through the environment, these ARGs should lead to particularly high sample AMR, although our samples had low richness of ARGs [35]. Wild boars had a higher richness of resistance genes than Tibetan pigs from Kangding farms, although its abundance of ARGs was the lowest among all tested samples (Fig. 2a, b). The wild boars used in this study were captured from the mountains near Nanchang City, Jiangxi Province, which is a densely populated city. A previous study showed that human activities have a huge impact on the distribution of antimicrobials and ARGs in the environment [11]. However, Tibetan pigs from KD-3800, KD-3480, and KD-1400 were raised in a rural region with a sparse human population.

Limited numbers of ARGs were identified as their integrated bacterial species. This would be explained by the limited reference genomes of bacterial species in the NR database. It should also be noted that the method used for identifying the bacterial species carrying these ARGs only suits for those ARGs integrated in the genomes of bacteria, but not for AMR traveling on mobile elements. *Escherichia coli* harbored the largest number of ARGs. Several previous studies reported *Escherichia coli* strains showing multidrug resistance in a variety of environments [62–65]. *Escherichia coli* has also been reported as a reservoir for fluoroquinolone and macrolide resistance genes, and the level of resistance has increased in recent years [66, 67]. We found that the ARGs not only related to these two drugs but also to peptide antibiotics which were particularly abundant and prevalent in *Escherichia coli*.

We found that the bacterial species that have been reported to show multi-drug resistance were the main carriers of ARGs (Fig. 5). Most of these bacteria are pathogens in humans. For instance, a strain of *Staphylococcus aureus* (ST398) resisting methicillin has been identified in both pigs and pig farmers in many countries [68], indicating the risk of these ARGs to both human and pigs. Some ARGs were carried by multiple bacteria. For example, *tet(W/N/W)* and *adeF* were detected in more than three bacterial species, indicating horizontal gene transfer occurring between bacterial species. The *tet(W/N/W)* demonstrating mobility was firstly reported in a Chinese pig manure sample in 2016; the gene encodes a mosaic ribosomal protection [51]. The *adeF* gene was reported to confer multidrug resistance by overexpression of the resistance-nodulation-cell division (RND) pump AdeFGH in *Acinetobacter baumannii* [50]. We found that *adeF* had a large number of homologous sequences detected in different bacteria (Additional file 11: Table S10), including *Acinetobacter baumannii*, *Escherichia coli*, and *Bacteroides stercoris*. Moreover, *adeF* was found in *Akkermansia muciniphila* and *Bacteroides fragilis,* which are potential probiotics (Fig. 5)*.* Several other potential probiotics were also identified as carrying resistance genes. This suggests that we should be careful in developing potential probiotics. Because of the high cost of metagenomic sequencing to evaluate the pollution of ARGs, we selected subsets of ARGs as the indicators predicting the abundance and richness of ARGs in environmental samples. The results from both discovery and validation datasets confirmed their high accuracy of prediction. These genes provided useful markers for evaluating the pollution level of ARGs by quantitative PCR.

## Conclusions

This study systematically investigated the diversity and distribution of fecal resistome in pigs raised under different rearing modes. The use of fecal samples from wild boars and Tibetan pigs from a sparsely populated region allowed us to evaluate the pollution level of ARGs under different rearing modes. In addition, we found significant differences in ARG profiles between cecum lumen and fecal samples and between piglets and adult pigs, suggesting that different sample sources should be used in evaluating the pollution of ARGs. In particular, the bacterial species integrated ARGs in their genomes were identified. We further selected a subset of ARGs as indicators for predicting the abundance and/or richness of ARGs. This should facilitate the evaluation of ARG pollution using simplified methods (e.g., qPCR). However, more comprehensive samples should be used to evaluate the overall pollution of ARGs in the pig industry. The results of this study provide a primary overview of ARG profiles in various farms under different rearing modes in China, and the data provide a reference for optimizing the use of antimicrobials and evaluating risks from ARGs in pig farms.

## Supplementary Information

**Additional file 1: Figure S1.** Identification of outlier samples before further analysis. a Violin plot for the ARGs abundance of 433 samples. b Comparison of the richness of gut microbiota between normal and outlier samples of F6 mosaic population. c Comparison of the abundance of AMR classes between normal and outlier samples in the F6 mosaic population. **Figure S2.** The composition and abundance of each AMR class in each population. **Figure S3.** The abundance and richness of 69 AMR classes. **Figure S4.** The composition and abundance of each resistance mechanism in each population. **Figure S5.** The prevalence and average abundance of 349 ARGs in all tested samples. **Figure S6.** The percentage of the abundance of core ARGs in the total abundance of all ARGs in each population. **Figure S7.** The effect of gender on the abundance (a) and the richness (b) of ARGs. **Figure S8.** Host genetic effect on the abundance (a) and the richness (b) of ARGs. **Figure S9.** Principal coordinate analysis (PCoA) based on Bray-Curtis distance indicating the distinct resistome among different pig populations. **Figure S10.** The prevalence of 46 core ARGs in cecum lumen of Wild boars and pigs at the age of 240 days from NC-F6 farm, and feces samples of piglets at the age of 25 days from NC-F6 farm. **Figure S11.** Comparison of the abundance and richness of resistome between two ages, and between two gut locations. a-b Comparison of the abundance (a) and richness (b) of resistome between 25 (*n* = 10) and 240 days of age (*n* = 10) in pigs from NC-F6 farm. c Comparison of the abundance and the number of ARGs between cecum lumen (*n* = 6) and feces samples (*n* = 6) in Wild boars. d Comparison of the abundance and the number of ARGs between cecum lumen (*n* = 10) and feces samples (*n* = 10) in pigs from NC-F6 farm. **Figure S12.** The ARGs that had high prevalence in cecum lumen samples but absent in feces in pigs under antimicrobial selection pressure. **Figure S13.** The distribution of times achieving significance level in 1000 times of comparison analyses by randomly selecting six out of ten pigs for each time analysis for 41 resistance classes. **Figure S14.** Workflow for identifying ARGs and their host bacteria. **Figure S15.** The abundance of ten indicators selected for predicting the total abundance of ARGs in each of 425 feces samples from 425 adult pigs.

**Additional file 2: Table S1.** The samples used in this study.

**Additional file 3: Table S2.** Information of 349 unique antimicrobial resistance genes (ARGs).

**Additional file 4: Table S3.** The abundance and ARG number of 69 drug resistance classes.

**Additional file 5: Table S4.** FDR and P values from pair-wise comparisons among nine pig populations.

**Additional file 6: Table S5.** The abundance and α-diversity of antimicrobial resistance in each sample.

**Additional file 7: Table S6.** Drug resistance classes showing significantly different abundances between 25 and 240 days of age, and between cecum lumen and feces in NC-F6 pigs.

**Additional file 8: Table S7.** The 194 ORFs that were clustered into 128 unique ARGs and annotated to 24 bacterial species.

**Additional file 9: Table S8.** The prevalence and average abundance of 24 bacterial species in samples.

**Additional file 10: Table S9.** The detailed information of the indicators selected for predicting the pollution level of resistance genes.

**Additional file 11: Table S10.** The homologous sequences of selected indicators in the gene catalog constructed in this study and the bacteria carring these ARGs.

## Data Availability

All metagenomic sequencing data are available at China National GeneBank DataBase (CNGBdb) with accession code: CNP0000824 (https://db.cngb.org/search/project/CNP0000824/). The codes for metagenomic analysis, statistical analyses, and visualization are available at https://github.com/zhouyunyan/PigARGs.

## Abbreviations

AMR: Antimicrobial resistance
ARGs: Antimicrobial resistance genes
qPCR: Quantitative real-time polymerase chain reaction
ORF: Open reading frame
SD: Standard deviations
CARD: The Comprehensive Antibiotic Resistance Database
M-L-S: Macrolide-lincosamide-streptogramin antibiotics
FDR: False discovery rate
PCoA: Principal coordinate
