# Supplementary material for: Extensive metagenomic analysis of the porcine gut resistome to identify indicators reflecting antimicrobial resistance
Source: Microbiome. 2022 Mar 4;10:39. doi: 10.1186/s40168-022-01241-y (PMC8895625; doi:10.1186/s40168-022-01241-y)
Supplement: Supplementary file 2 — Additional file 1: Figure S1. Identification of outlier samples before further analysis. a Violin plot for the ARGs abundance of 433 samples. b Comparison of the richness of gut microbiota between normal and outlier samples of F6 mosaic population. c Comparison of the abundance of AMR classes between normal and outlier samples in the F6 mosaic population. Figure S2. The composition and abundance of each AMR class in each population. Figure S3. The abundance and richness of 69 AMR classes. Figure S4. The composition and abundance of each resistance mechanism in each population. Figure S5. The prevalence and average abundance of 349 ARGs in all tested samples. Figure S6. The percentage of the abundance of core ARGs in the total abundance of all ARGs in each population. Figure S7. The effect of gender on the abundance (a) and the richness (b) of ARGs. Figure S8. Host genetic effect on the abundance (a) and the richness (b) of ARGs. Figure S9. Principal coordinate analysis (PCoA) based on Bray-Curtis distance indicating the distinct resistome among different pig populations. Figure S10. The prevalence of 46 core ARGs in cecum lumen of Wild boars and pigs at the age of 240 days from NC-F6 farm, and feces samples of piglets at the age of 25 days from NC-F6 farm. Figure S11. Comparison of the abundance and richness of resistome between two ages, and between two gut locations. a-b Comparison of the abundance (a) and richness (b) of resistome between 25 (n = 10) and 240 days of age (n = 10) in pigs from NC-F6 farm. c Comparison of the abundance and the number of ARGs between cecum lumen (n = 6) and feces samples (n = 6) in Wild boars. d Comparison of the abundance and the number of ARGs between cecum lumen (n = 10) and feces samples (n = 10) in pigs from NC-F6 farm. Figure S12. The ARGs that had high prevalence in cecum lumen samples but absent in feces in pigs under antimicrobial selection pressure. Figure S13. The distribution of times achieving significance level in 1000 [file 40168_2022_1241_MOESM2_ESM.docx]

**
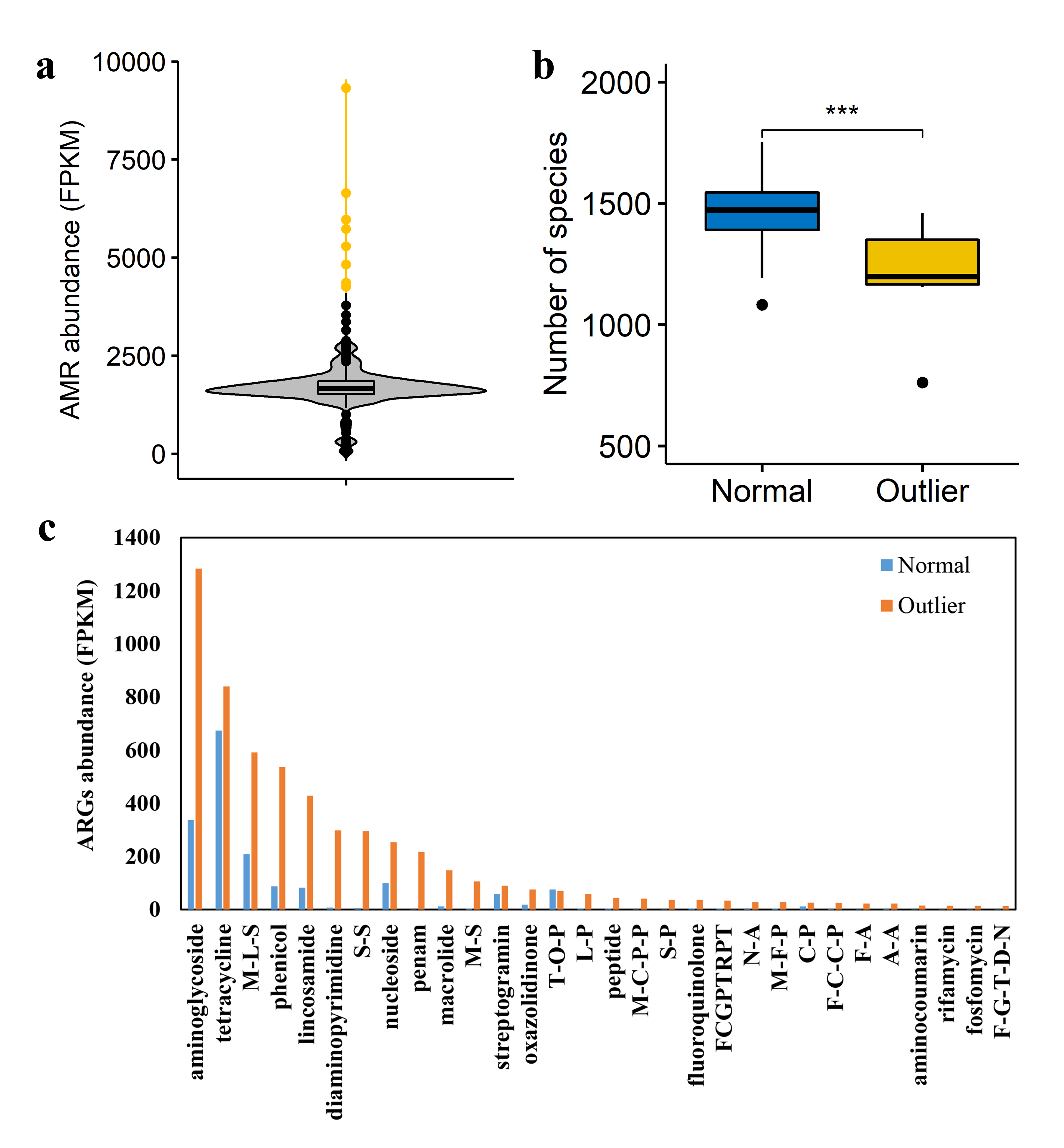
**

**Figure S1.** Identification of outlier samples before further analysis. **a** Violin plot for the ARGs abundance of 433 samples. Eight samples from F6 mosaic population were excluded (below/higher than the mean plus/minus 3 standard deviations, yellow) for the downstream analysis. **b** Comparison of the richness of gut microbiota between normal and outlier samples in the F6 mosaic population. ****P* < 0.001, Wilcoxon test. **c** Comparison of the abundance of AMR classes between normal and outlier samples in F6 mosaic population. Note that the corresponding full names of the abbreviated antimicrobials classes used in all figures of this study are presented in Additional file 4: Table S3.

**
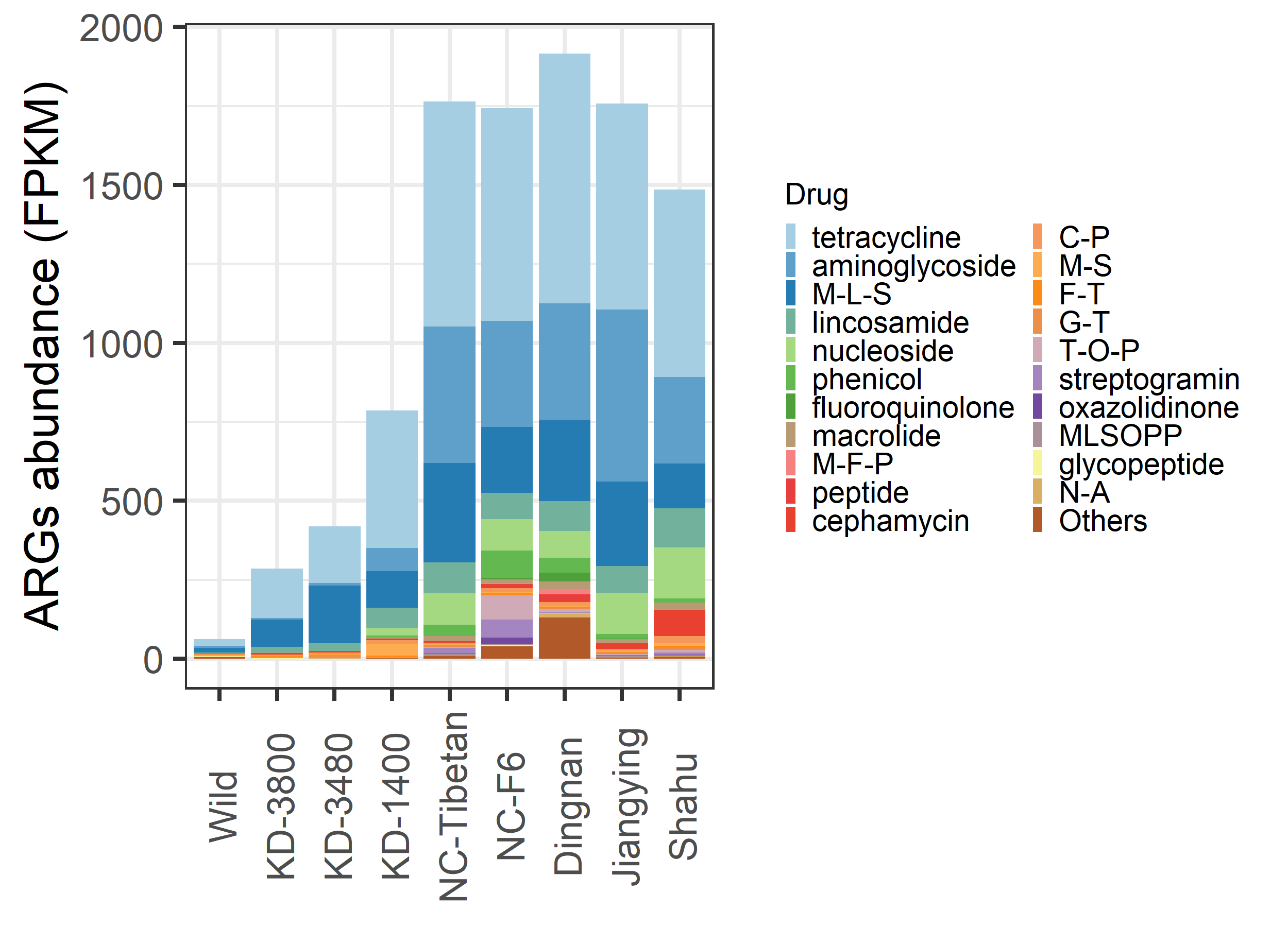
**

**Figure S2.** The composition and abundance of each AMR class in each population.

**
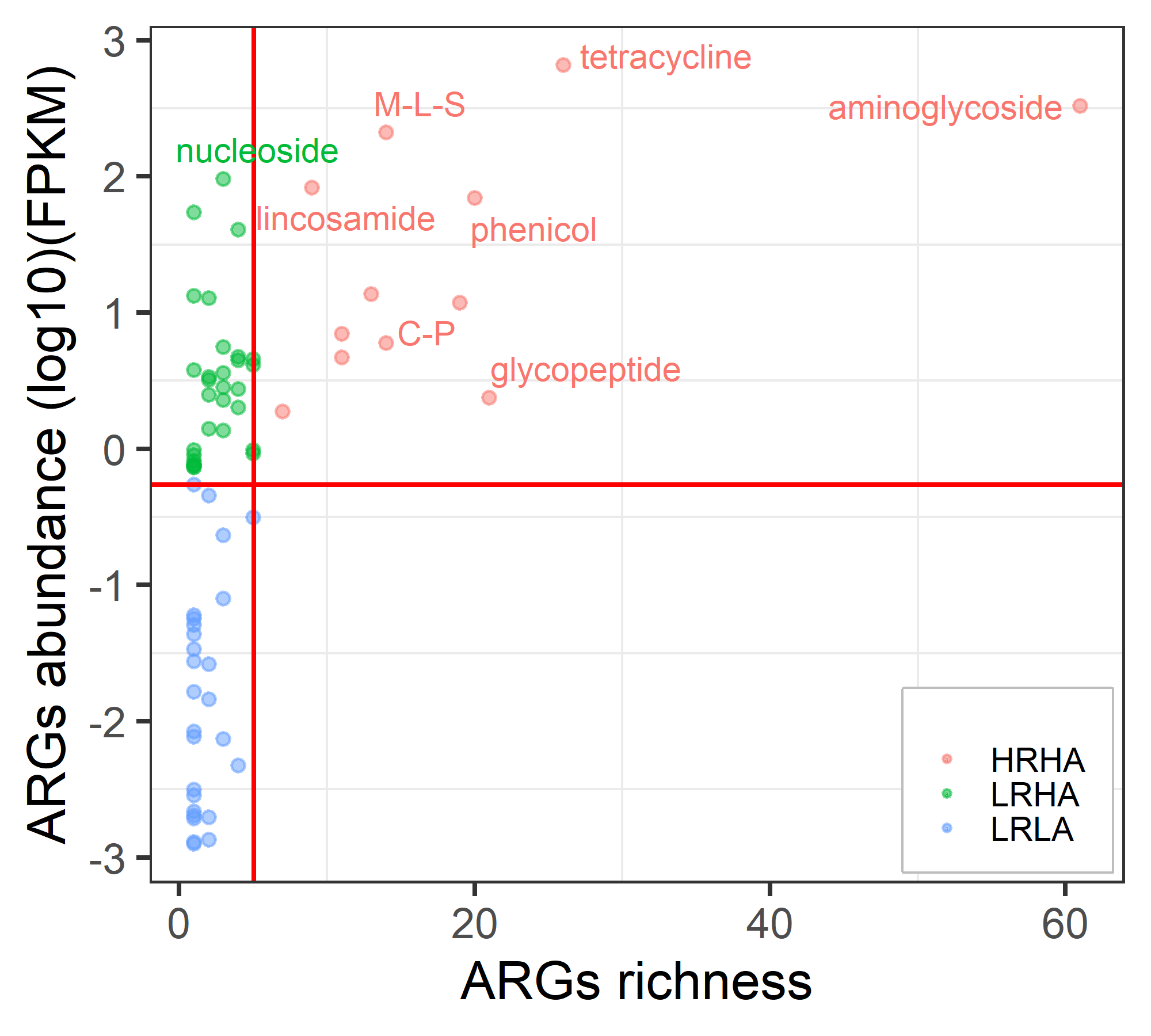
**

**Figure S3.** The abundance and richness of 69 AMR classes. The abundance was log-transformed. The horizontal and vertical red lines on the plot indicate the average abundance and richness of AMR classes, respectively. AMR classes are colored according to their abundance and richness. HRHA (high richness and high abundance, red), LRHA (low richness but high abundance, green) and LRLA (low richness and low abundance, blue). Top five AMR classes of the abundance and richness were annotated on the plot.


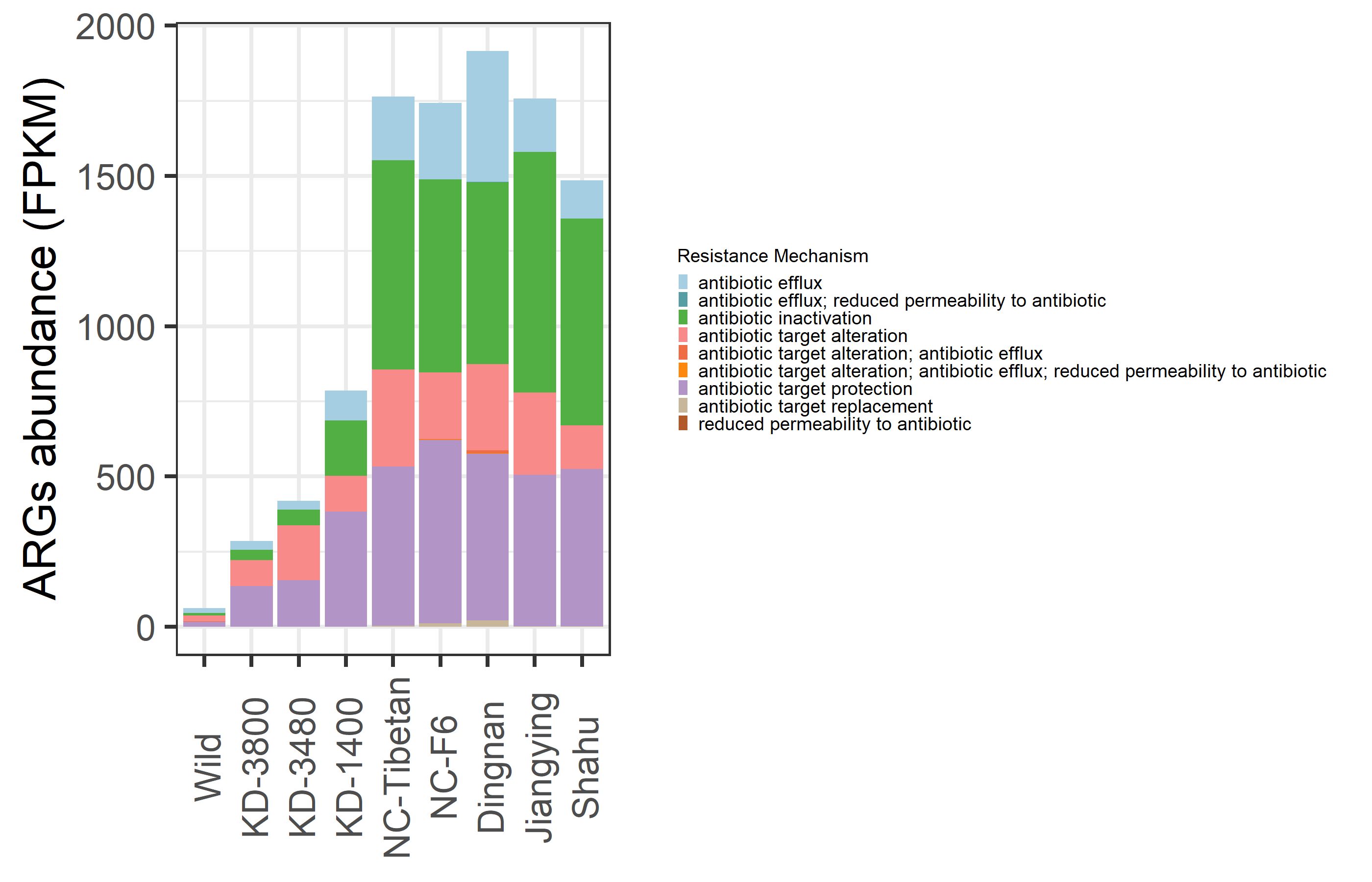


**Figure S4.** The composition and abundance of resistance mechanisms in each population.


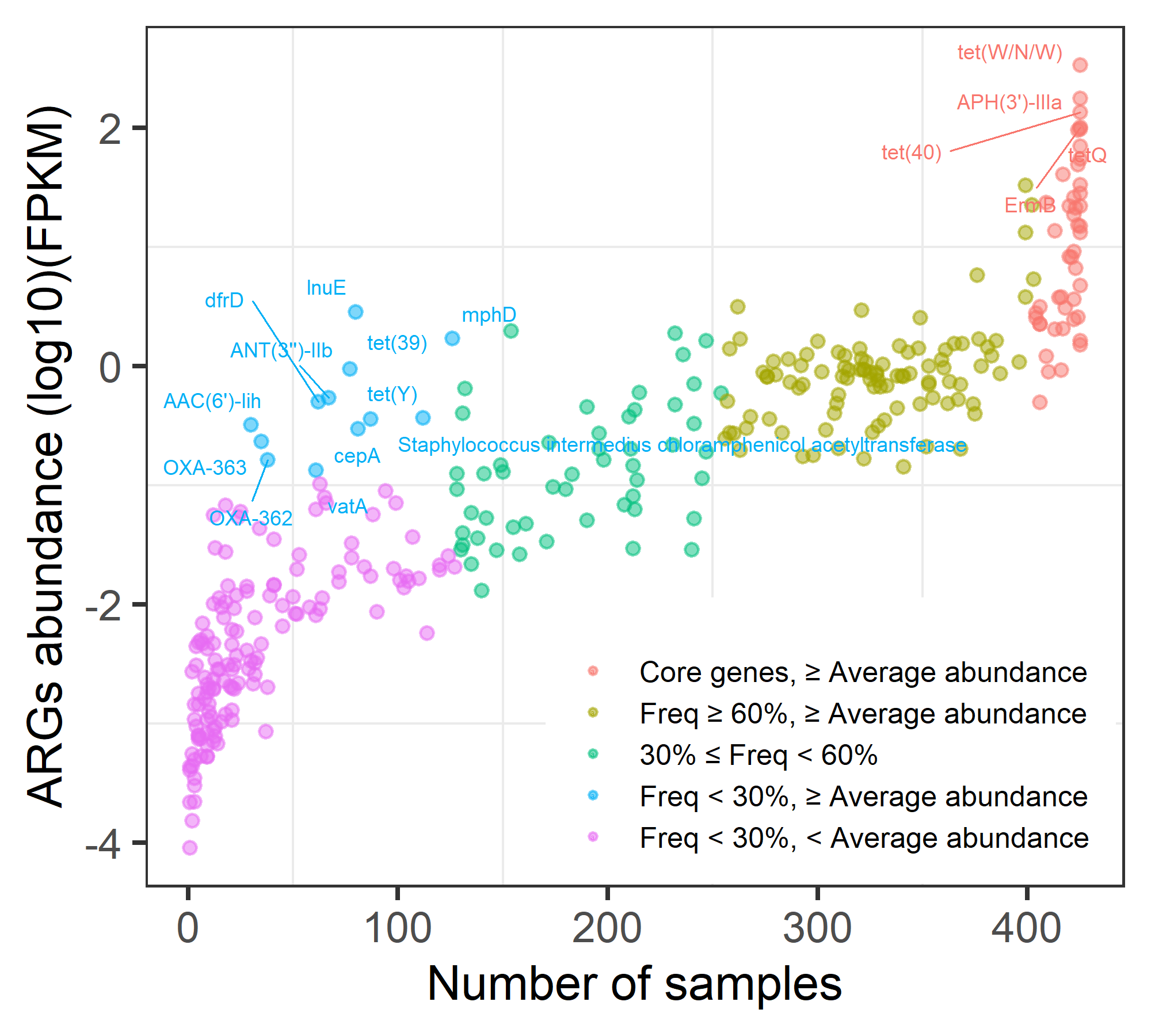


**Figure S5.** The prevalence and average abundance of 349 ARGs in all tested samples. The ARGs existing in at least 95% of tested samples (n≥404) was defined as core ARGs. The top five ARGs in the abundance, the ARGs showing low prevalence (Freq <30%) but having high abundance (>Average abundance) in the detected samples were annotated with text on the plot.


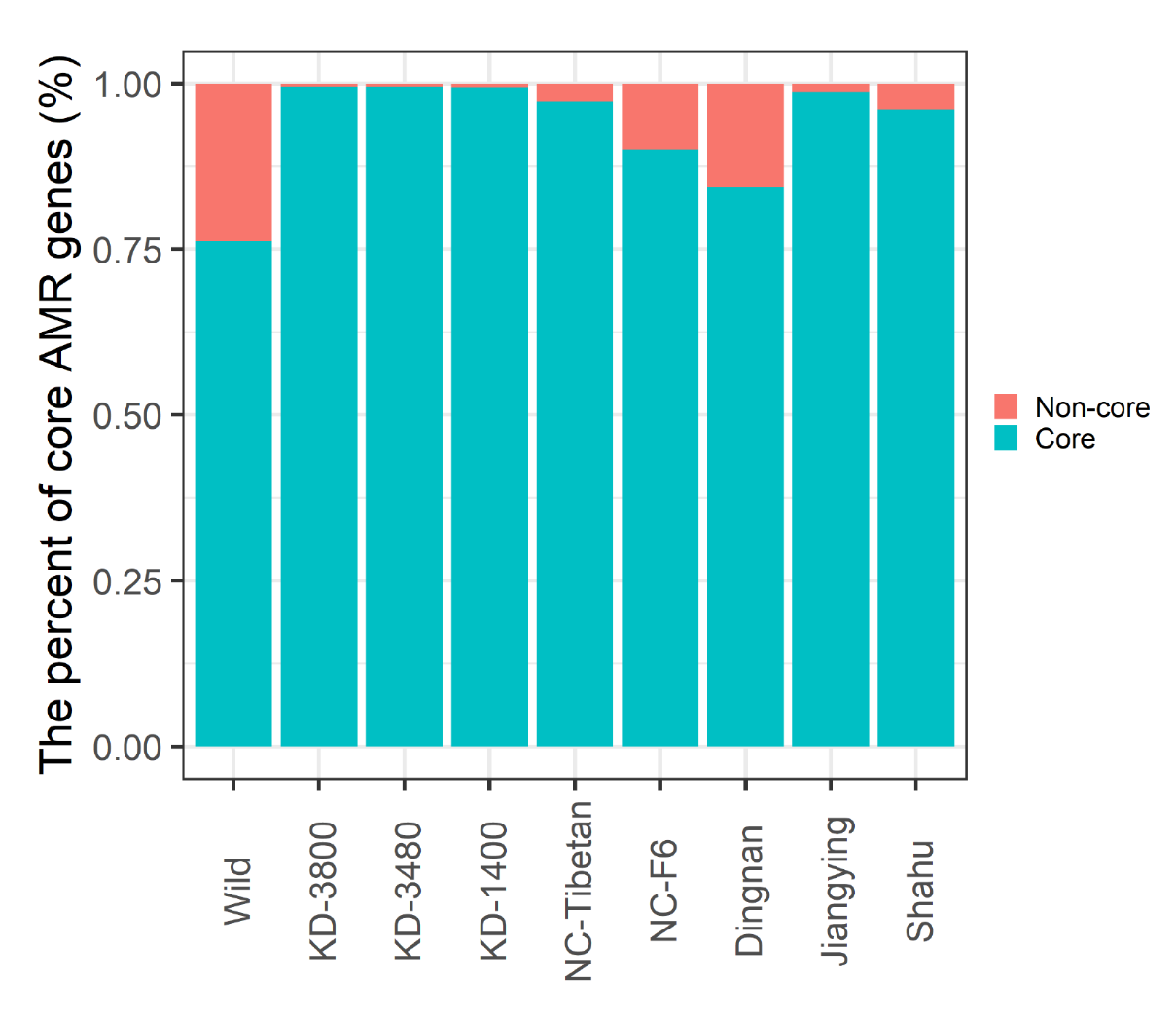


**Figure S6.** The percentage of the abundance of core ARGs in the total abundance of all ARGs in each population. The percentage was the average percentage of all pigs in each farm. The genes exited in more than 95% samples (n≥404) were defined as core ARGs.


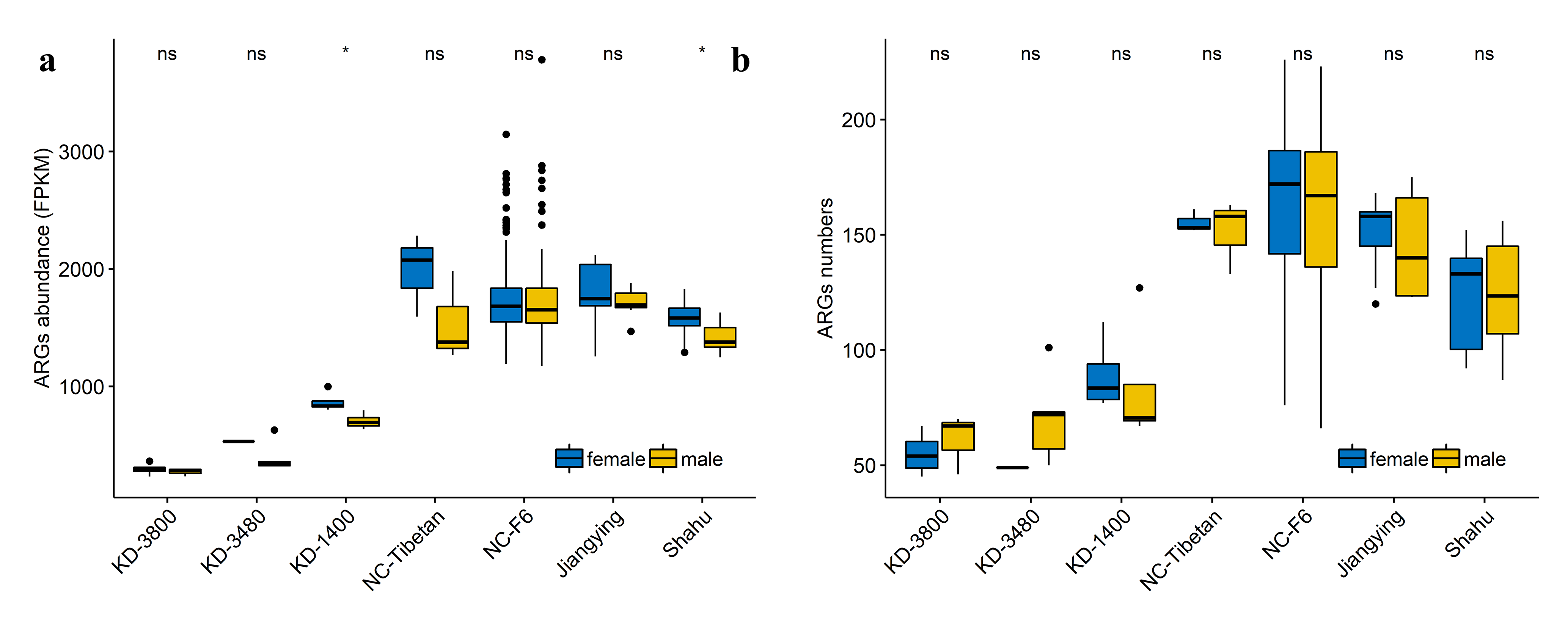


**Figure S7.** The effect of gender on the abundance (**a**) and the richness (**b**) of ARGs. ns, non-significance (*P*≥0.05); **P* < 0.05, Wilcoxon test was used to the comparison analysis. Dingnan farm (only sows) and Wild boars (the gender information for several Wild boars was unknown) were not included in this analysis.


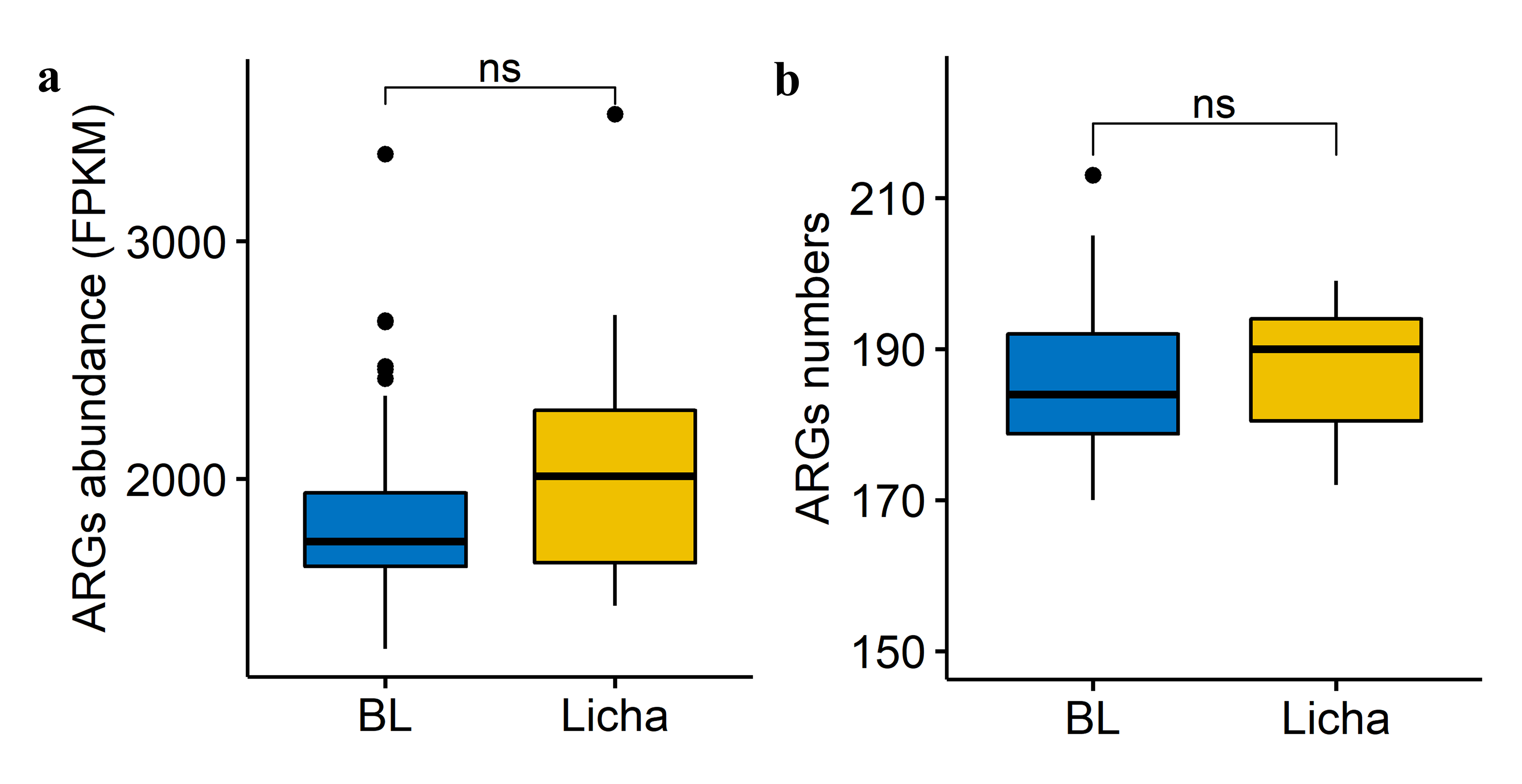


**Figure S8.** Host genetic effect on the abundance (**a**) and the richness (**b**) of ARGs. The dataset from Licha (n = 14) and Berkshire × Licha line (n = 49) were used. There was no significant difference in both abundance and richness of ARGs. ns, non-significance (*P*≥0.05), The analysis was performed by Wilcoxon test.


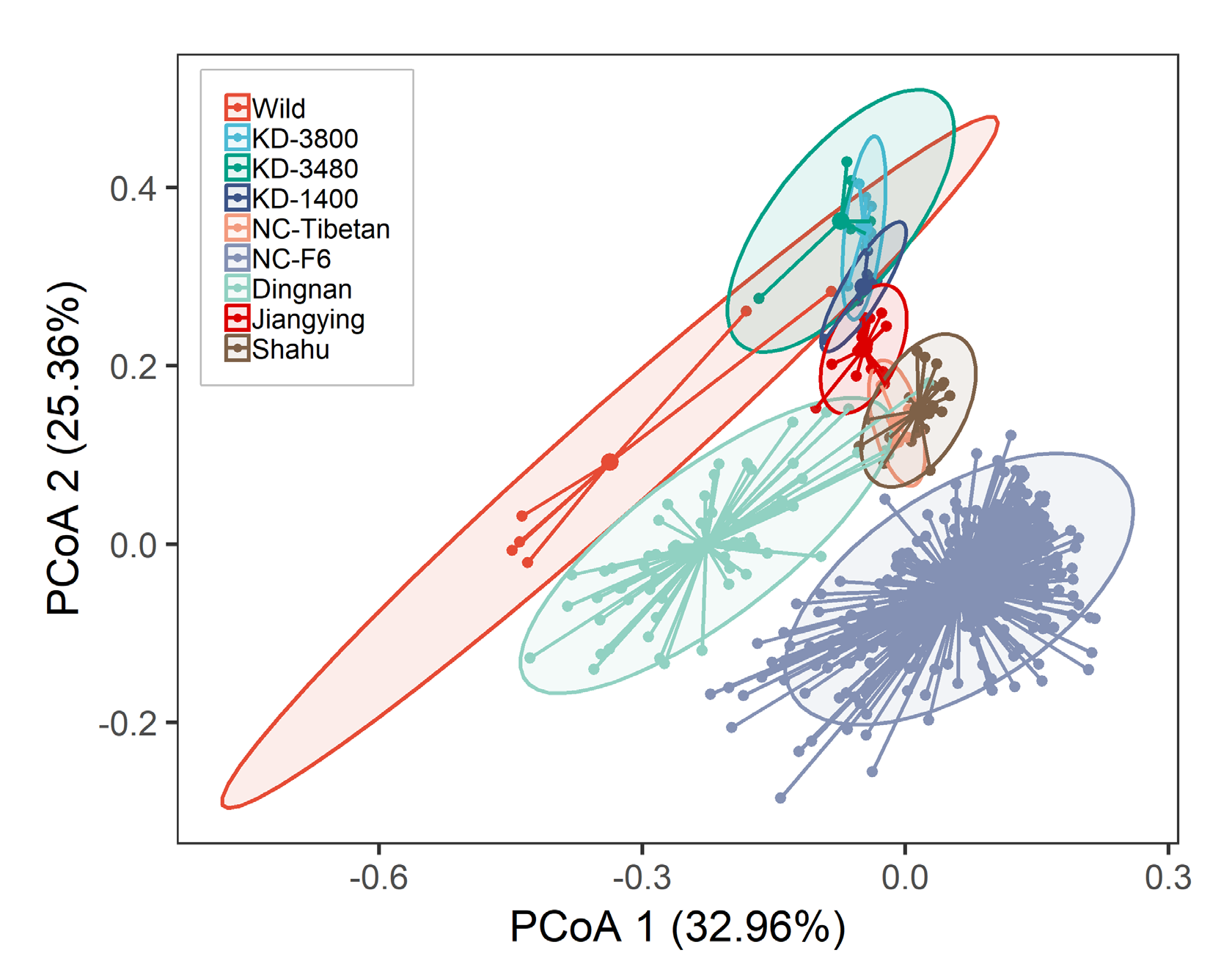


**Figure S9.** Principal coordinate analysis (PCoA) based on Bray-Curtis distance indicating the distinct resistome among different pig populations. The abundance matrices of ARGs (FPKM) were Hellinger transformed.


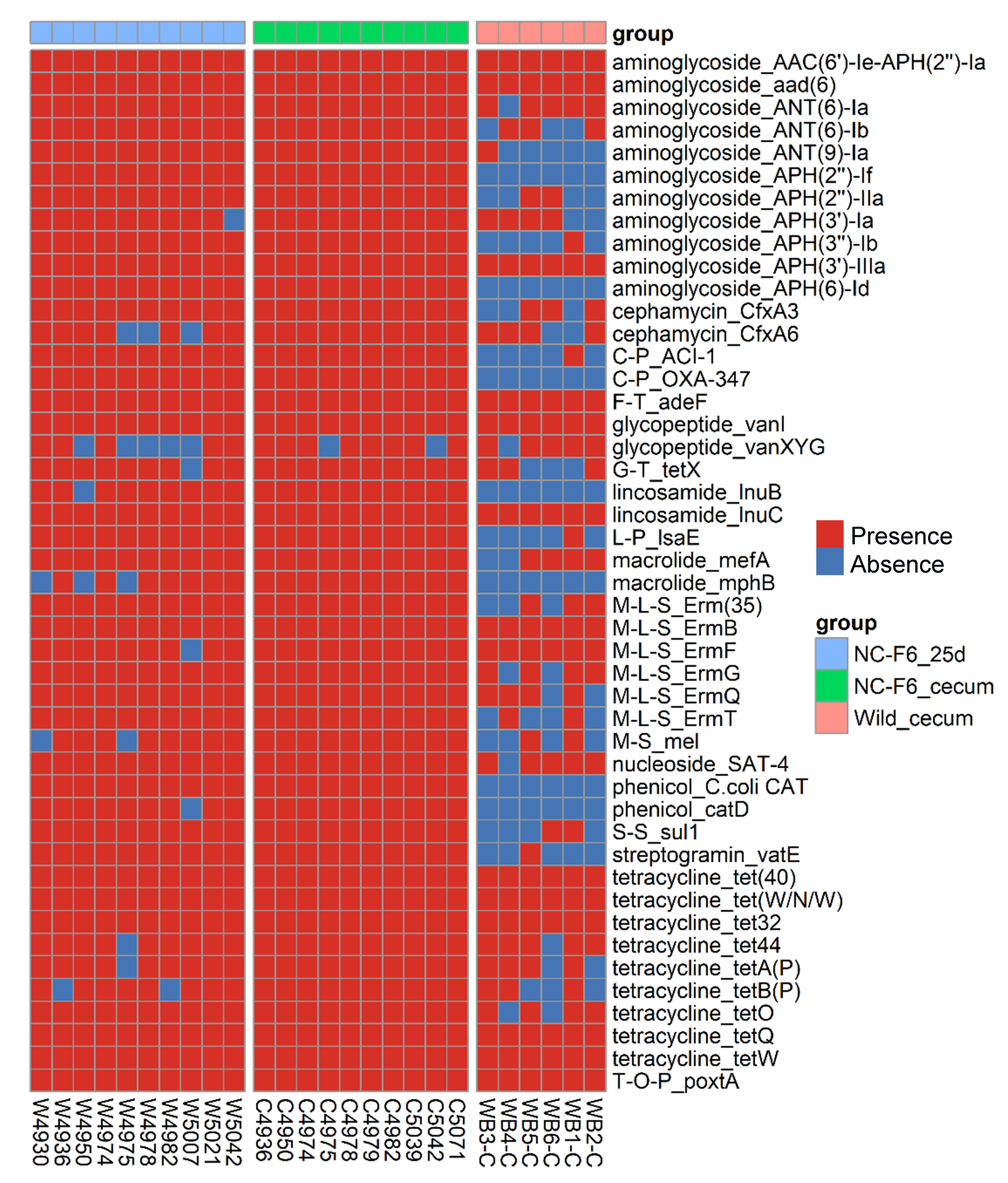


**Figure S10.** The prevalence of 46 core ARGs in cecum lumen of Wild boars and pigs at the age of 240 days from NC-F6 farm, and feces samples of piglets at the age of 25 days from NC-F6 farm. W + numbers: feces samples at the age of 25 days from NC-F6 pigs, C + numbers: cecum samples at the age of 240 days from NC-F6 pigs , and WB + numbers + C: cecum samples from Wild boars. Same numbers represent samples from same pig.


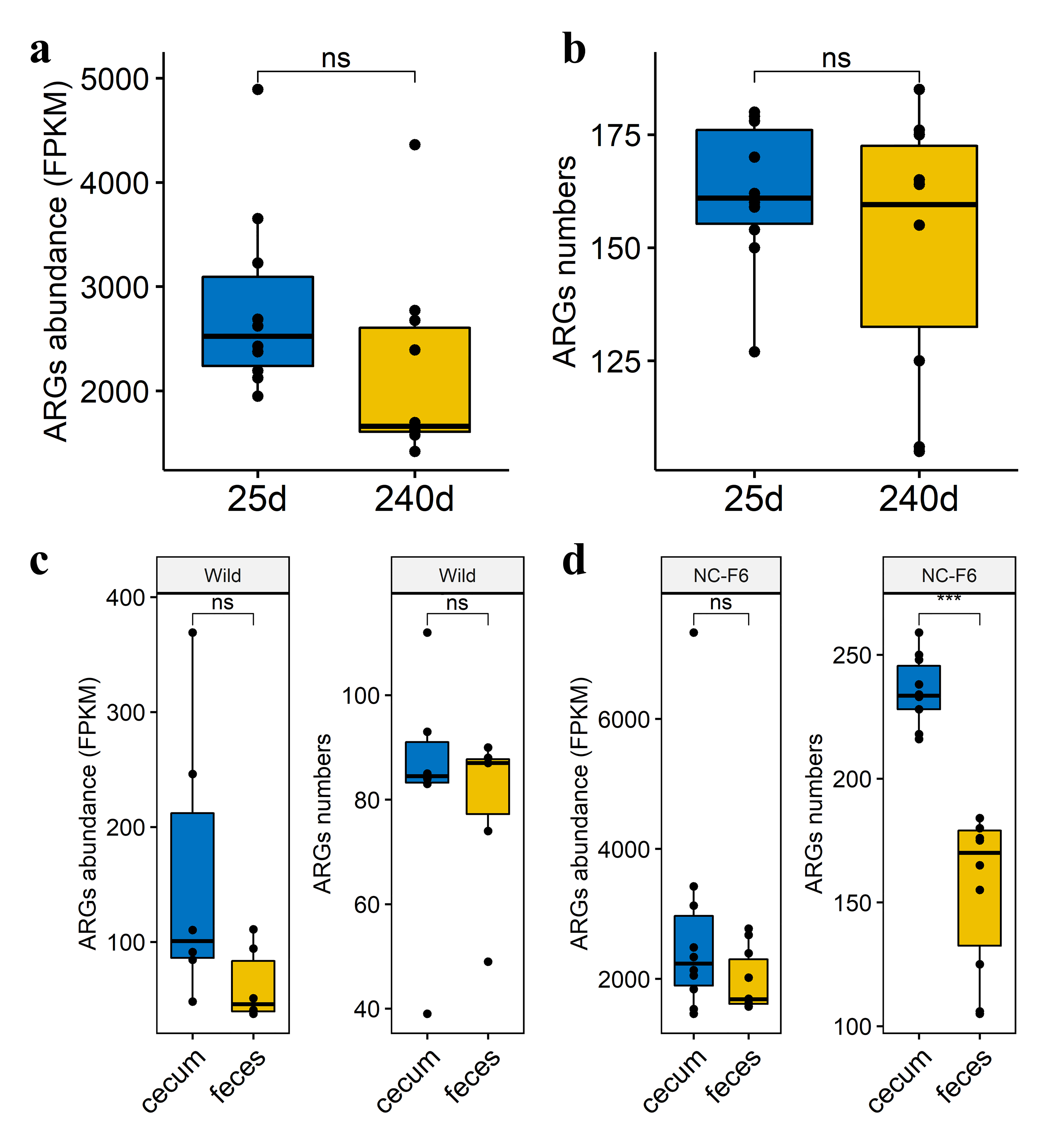


**Figure S11.** Comparison of the abundance and richness of resistome between two ages, and between two gut locations. **a-b.** Comparison of the abundance (**a**) and the number (**b**) of antibiotic genes (ARGs) between 25 (n = 10) and 240 days of age (n = 10) in pigs from NC-F6 farm. ns, non-significance (*P* ≥ 0.05). The comparison was performed by Wilcoxon test. **c.** Comparison of the abundance and the number of ARGs between cecum lumen (n = 6) and feces samples (n = 6) in Wild boars. **d.** Comparison of the abundance and the number of ARGs between cecum lumen (n = 10) and feces samples (n = 10) in pigs from NC-F6 farm. ****P* < 0.001


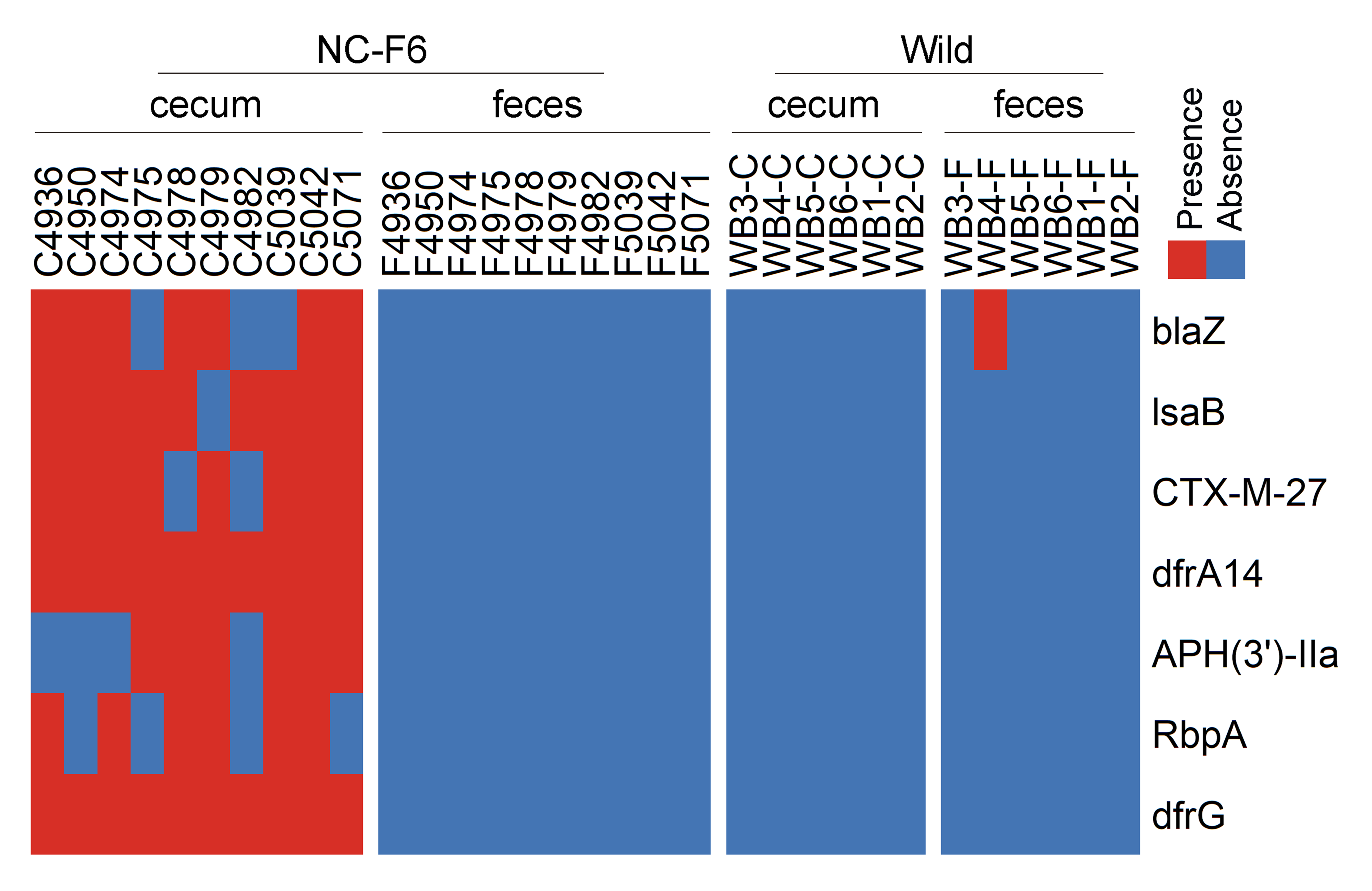


**Figure S12.** The ARGs that had high prevalence in cecum lumen samples but absent in feces in pigs under antimicrobial selection pressure. Seven ARGs were enriched in cecum lumen samples of pigs from NC-F6 farms. However, these genes were absent in both cecum lumen and feces samples of Wild boars. The x-axis shows the sample IDs. C + numbers: cecum samples at the age of 240 days from from NC-F6 pigs , F + numbers: feces samples at the age of 240 days from NC-F6 pigs, WB + numbers + C: cecum samples from Wild boars, and WB + numbers + C: feces samples from Wild boars. Same numbers represent samples from same pig.


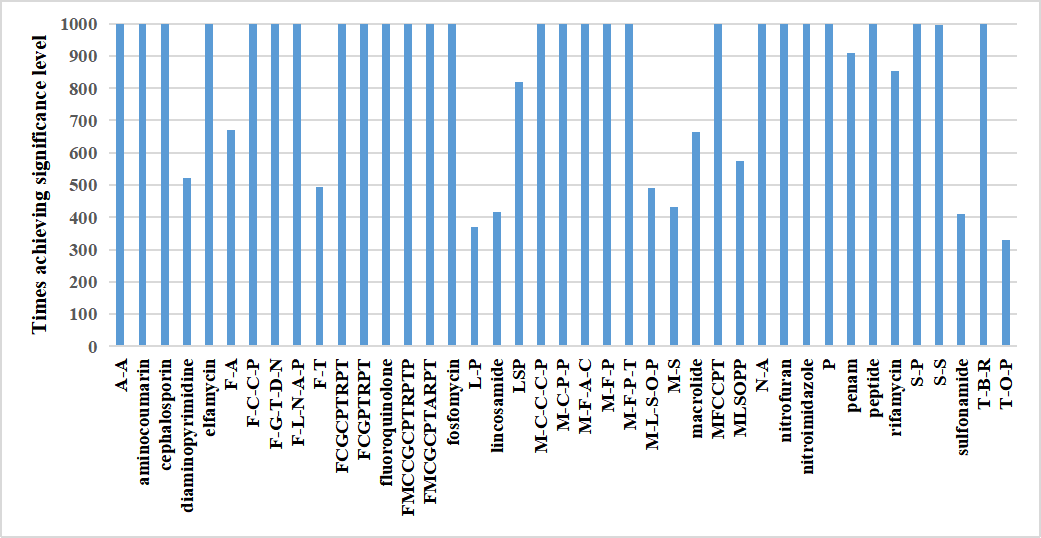


**Figure S13.** The distribution of times achieving significance level in 1000 times of comparison analyses by randomly selecting six out of ten pigs for each time analysis for 41 resistance classes. The 41 resistance classes were shown differential abundance between cecum lumen and fecal samples in ten experimental pigs from NC-F6 population.


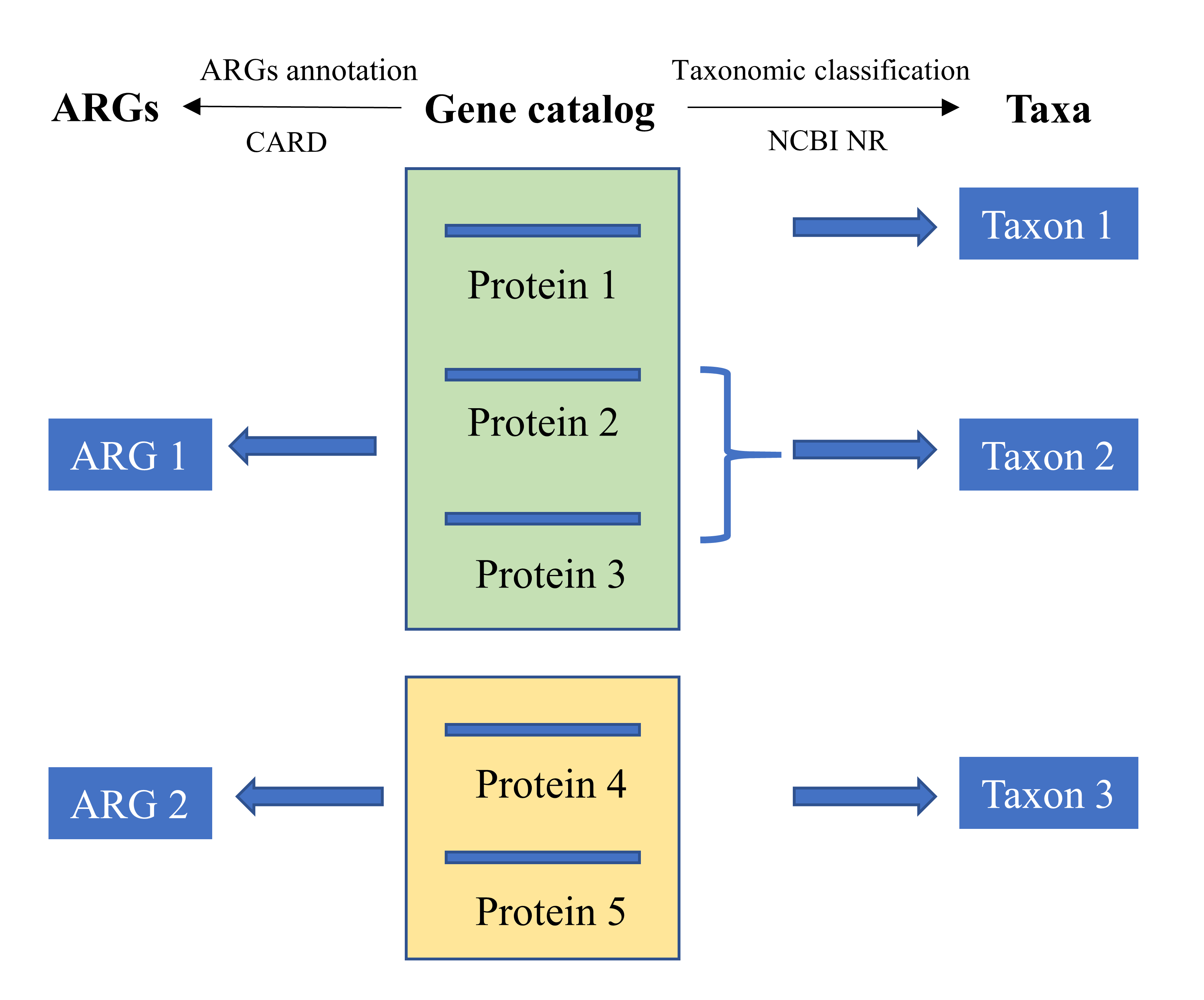


**Figure S14.** Workflow for identifying ARGs and their host bacteria. Proteins (ORFs) were annotated to CARD database to identify ARGs and mapped to NCBI NR database to annotate the bacteria. If an ORF was identified as an ARG and annotated to a bacterium, we considered that this bacterium was the host bacterium of this ARG. Taxonomic classification was performed by aligning protein sequence to the NCBI NR database (Version: 2019-04). The ARGs were identified by alignment against the Comprehensive Antibiotic Resistance Database (CARD). Multiple protein sequences may be annotated to the one type of resistance gene or microbial taxon because of the gene catalogue was generated by clustering at the 95% identity at protein level.

**
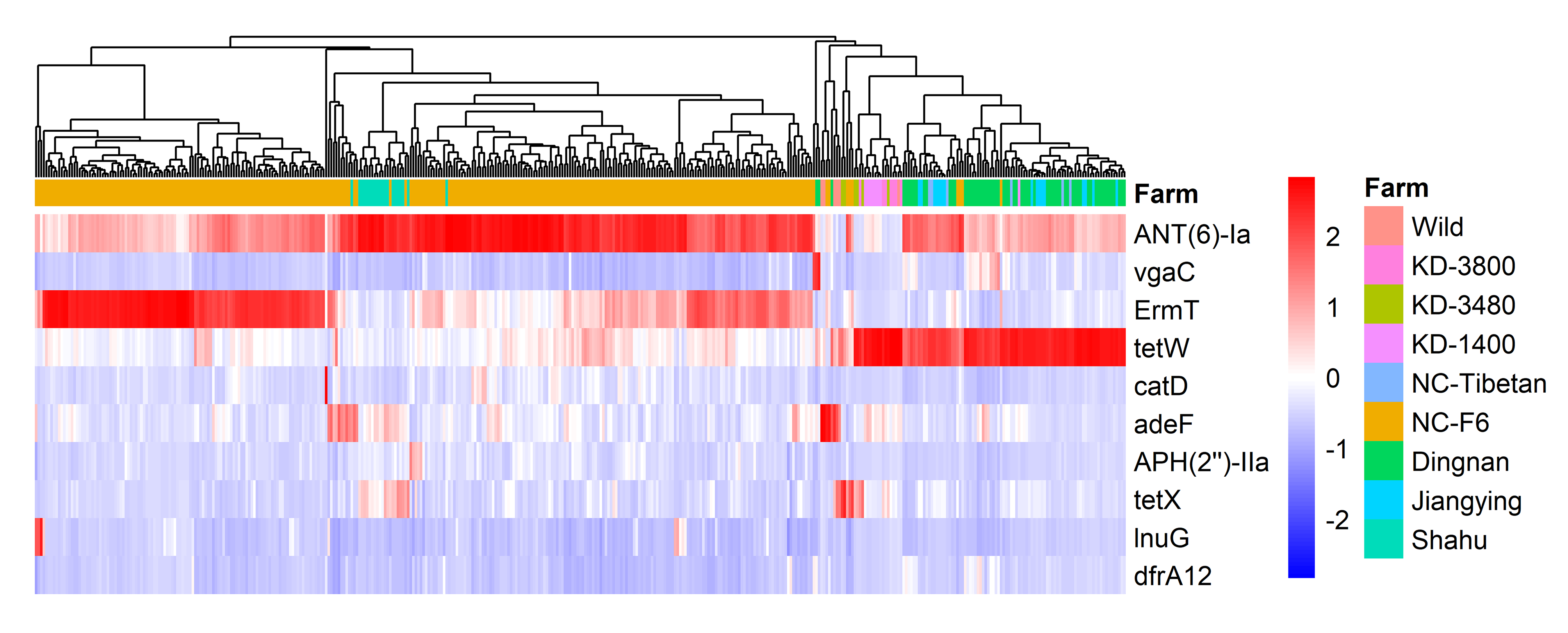
**

**Figure S15.** The abundance of ten indicators selected for predicting the total abundance of ARGs in each of 425 feces samples from 425 adult pigs.
